# Fluoride detoxification in tea plants depends on aluminum and localization in the epidermis

**DOI:** 10.1093/plphys/kiag077

**Published:** 2026-02-21

**Authors:** Chenyu Zhang, Paula Pongrac, Katarina Vogel-Mikuš, Alessandra Gianoncelli, Valentina Bonanni, Matjaž Kavčič, Žiga Šmit, Zdravko Rupnik, Primož Vavpetič, Mark G M Aarts, Antony van der Ent

**Affiliations:** Laboratory of Genetics, Wageningen University and Research, Droevendaalsesteeg 1, 6708 PB Wageningen, The Netherlands; Key Laboratory of Biology, Genetics and Breeding of Special Economic Animals and Plants, Ministry of Agriculture and Rural Affairs, Tea Research Institute of the Chinese Academy of Agricultural Sciences, Hangzhou 310008, China; Department of Biology, Biotechnical Faculty, University of Ljubljana, Jamnikarjeva 101, 1000 Ljubljana, Slovenia; Department of Low and Medium Energy Physics, Jožef Stefan Institute, Jamova 39, 1000 Ljubljana, Slovenia; Department of Biology, Biotechnical Faculty, University of Ljubljana, Jamnikarjeva 101, 1000 Ljubljana, Slovenia; Department of Low and Medium Energy Physics, Jožef Stefan Institute, Jamova 39, 1000 Ljubljana, Slovenia; Elettra–Sincrotrone Trieste, Strada Statale 14-km 163,5 in AREA Science Park, Trieste-Basovizza 34149, Italy; Elettra–Sincrotrone Trieste, Strada Statale 14-km 163,5 in AREA Science Park, Trieste-Basovizza 34149, Italy; Department of Low and Medium Energy Physics, Jožef Stefan Institute, Jamova 39, 1000 Ljubljana, Slovenia; Department of Physics, Faculty of Mathematics and Physics, University of Ljubljana, Jadranska 19, 1000 Ljubljana, Slovenia; Department of Low and Medium Energy Physics, Jožef Stefan Institute, Jamova 39, 1000 Ljubljana, Slovenia; Department of Physics, Faculty of Mathematics and Physics, University of Ljubljana, Jadranska 19, 1000 Ljubljana, Slovenia; Department of Low and Medium Energy Physics, Jožef Stefan Institute, Jamova 39, 1000 Ljubljana, Slovenia; Department of Low and Medium Energy Physics, Jožef Stefan Institute, Jamova 39, 1000 Ljubljana, Slovenia; Laboratory of Genetics, Wageningen University and Research, Droevendaalsesteeg 1, 6708 PB Wageningen, The Netherlands; Laboratory of Genetics, Wageningen University and Research, Droevendaalsesteeg 1, 6708 PB Wageningen, The Netherlands

## Abstract

Tea (*Camellia sinensis*) is a hyperaccumulator of both aluminum (Al) and fluorine (F). While the formation of Al-F complexes has been proposed as a key mechanism for F detoxification and accumulation in tea, excessive Al and especially fluoride (F^−^) pose health risks to humans. In this study, we tested the hypothesis that Al^3+^ mitigates F^−^-induced ionomic imbalance and that Al and F are spatially colocalized within tea tissues. Tea plants were grown hydroponically under different Al^3+^ and F^−^ treatments, and elemental distributions were investigated using micro-particle-induced gamma-ray emission (micro-PIGE), micro-particle-induced X-ray emission (micro-PIXE), and low-energy X-ray fluorescence (LEXRF) analyses. Tea plants were highly sensitive to F^−^ treatment, exhibiting leaf crinkling, chlorosis, and marginal necrosis; however, the addition of Al^3+^ markedly alleviated these symptoms by reducing F^−^ translocation to the shoots. Ionomic profiling revealed that F^−^ supply increased manganese (Mn) accumulation in both leaves and roots, whereas Al^3+^ supplementation mitigated F^−^-induced Mn toxicity. Micro-PIGE mapping revealed co-localization of Al and F in leaf margins, particularly along fourth- and fifth-order veins. LEXRF analysis further showed that Al and F colocalized in the epidermis of leaves and roots, but not in the xylem of petiole or midribs. In root cortical cells, Al and magnesium (Mg) colocalized. These findings demonstrate that F^−^ detoxification in tea is Al^3+^-dependent and occurs through the formation of Al-F complexes in the epidermis, providing a spatial framework for future mechanistic studies on Al-F interactions in tea.

## Teaser

Aluminum reduces fluoride toxicity in tea plants by limiting fluoride movement to shoots and forming Al-F complexes in leaf and root epidermis, explaining how plants detoxify these elements.

## Introduction

Tea (*Camellia sinensis*) is predominately cultivated in tropical and subtropical regions, where acidic soils are widespread and soluble forms of aluminum (Al), particularly Al^3+^, Al(OH)^2+^, and Al(OH)_2_^+^, are highly phytotoxic to most plant species (Blamey et al. 1991). Nevertheless, tea exhibits exceptional tolerance to Al toxicity and is recognized as an Al hyperaccumulator, capable of accumulating up to 30,000 μg·g^−1^ Al in its leaves under natural conditions (Matsumoto et al. 1976). The presence of Al has been reported to promote tea plant growth, while Al depletion severely impairs root development, suggesting that tea plants not only tolerate and accumulate Al but also require it for normal growth (Sun et al. 2020; Yamashita et al. 2020). In addition to Al, tea plant is also known to accumulate fluoride (F^−^), the natural ionic form of fluorine (F), with concentrations in the leaves, particularly in older leaves reaching 300 to 1,000 μg·g^−1^ (Lu et al. 2004). Fluorine is considered non-essential to plants, and the concentration of F^−^ in plants is typically below 20 μg·g^−1^ (van der Ent et al. 2021). Some tea products, such as brick tea made from mature leaves and stems, contain F^−^ concentrations 2 to 4 times higher than those found in green or black tea (Fung et al. 1999). Excessive consumption of such products has been associated with an increased risk of fluorosis (Cao et al. 1997; Wen et al. 2022). As Alzheimer disease is linked to the Al^3+^ and F^−^ content in the human brain, the high concentrations of Al^3+^ and F^−^ in tea has garnered special attention (Wong et al. 2003; Exley and Clarkson 2020). Previous studies have explored the mechanisms underlying Al and F interaction in tea and revealed that tea plants are highly sensitive to F^−^ toxicity in the absence of Al^3+^. Aluminum has therefore been proposed to alleviate F^−^ toxicity and promote F uptake by forming Al-F complexes (Ruan et al. 2003; Yang et al. 2016).

Given the high accumulation of both Al and F in tea leaves, understanding their cellular and subcellular localization is of great interest. Previous studies employing various techniques, such as electron microprobe X-ray (EMX), low-energy X-ray fluorescence (LEXRF), X-ray fluorescence microscopy (XRM), and micro-particle-induced X-ray emission (micro-PIXE) have demonstrated that Al is primarily localized in the epidermal cell walls of tea leaves, with a preferential accumulation in the upper epidermis (Matsumoto et al. 1976; Tolrà et al. 2011; Pongrac et al. 2020; van der Ent et al. 2020). Theoretically, the strong chemical affinity between Al and F suggests their co-localization within leaf tissues (Castiglione et al. 1999). However, a micro-particle–induced gamma-ray emission (micro-PIGE) study reported that F is uniformly distributed in the cytoplasm of upper epidermal cells and highly concentrated in the lower epidermis, although the resolution of their image was limited to 400 μm × 400 μm (Yoshida et al. 2013). Using osmotic lysis and differential centrifugation methods, Gao et al. (2014) found that only 18.9% of F^−^ was present in the cell wall fraction, whereas 71.8% was detected in the soluble fraction of tea leaves. In contrast, Li et al. (2025) reported that more than 80.8% of total F^−^ accumulated in the cell wall. These contrasting results raised the possibility that tea plants may spatially separate Al-F complexes through binding with other ligands, such as catechins or organic acids (Morita et al. 2004). Alternatively, the reported distributions may require re-evaluation using high-resolution imaging techniques.

Since the ability of tea plants to accumulate Al^3+^ and F^−^ was reported in the 1930s (Harrison 1949), this species has been the focus of extensive research (Ding et al. 2021). Numerous studies have reported a strong correlation between Al and F in terms of both concentration and physiological responses (Ruan and Wong 2001; Yang et al. 2016). However, none to our knowledge have simultaneously investigated their spatial localization within plant tissues. In the present study, we aim to explore the spatial distribution of Al and F in the leaf, petiole, and root of tea plants using cutting-edge techniques: micro-PIGE, micro-PIXE, and LEXRF spectromicroscopy.

## Materials and methods

### Plant materials and culture conditions and aluminum and fluoride dosing

Seeds of *Camellia sinensis* var. *sinensis* (Onszaden, Wageningen, Netherlands) were mechanically scarified using sandpaper until the seed coats were completely removed and subsequently soaked in tap water for 3 days. The pretreated seeds were sown 2 cm below the surface in a moist medium composed of 50% perlite and 50% vermiculite and allowed to germinate for 5 to 7 weeks until seedlings developed 3 to 4 true leaves. Germinated seedlings were then transferred to polyethylene pots containing 4 L of half-strength Hoagland nutrient solution supplemented with 20 mmol·L^−1^ MES buffer (pH 5.5). The solution was continuously aerated, and 3 plants were grown per pot. The composition of the Hoagland nutrient solution is provided in Table S3. After 1 month of hydroponic growth, the seedlings were subjected to the following treatments: 250 μM aluminum chloride (AlCl_3_·6H_2_O; Al), 250 μM sodium fluoride (NaF; F), a combined treatment of 250 μM AlCl_3_·6H_2_O + 250 μM NaF (Al + F), and a control treatment without Al or F addition (CT). All nutrient solutions were adjusted to pH 4.2 and supplemented with a buffer composed of 53 mmol·L^−1^ sodium acetate and 147 mmol·L^−1^ acetic acid, and were refreshed every 2 weeks (Tolrà et al. 2011; Pongrac et al. 2020). During the treatment period, the pH of the solution was monitored at each nutrient replacement using a calibrated pH meter to ensure stability. The plants were grown in a controlled-environment chamber set to a 12-h-light/12-h-dark photoperiod, with a light intensity of 150 μmol m^−2^ s^−1^, relative humidity of 70%, and a constant temperature at 20 ± 2℃. After 2 months of treatment, tissues were collected from 4-month-old tea seedlings, including the tender leaves (TL; bud, first, second, and third leaves), mature leaves (ML; fourth to sixth leaves), and roots (10 cm from the apex). For the quantification experiment using MXRF, tea leaf samples were harvested manually, and the surface was gently wiped with deionized water to remove surface contaminants. Root samples were excised using scissors and thoroughly rinsed 3 times with deionized water. All samples were then oven-dried at 60 °C for 7 days before analysis. For the element mapping experiment, 4-month-old healthy tea seedlings subjected to Al and F treatments were carefully packaged and transported to the Biotechnical Faculty, University of Ljubljana. The mature leaves, petioles, and roots were chosen for further sample preparation and analysis.

### Determination of fluoride using ionic-specific electrode

Fluoride concentrations were determined using a F^−^ ion-selective electrode (perfectION Combination Fluoride Electrode, Mettler Toledo, the Netherlands) connected to a portable meter (SevenGo pro, Mettler Toledo, the Netherlands). The F analysis protocol followed the methods described in Zhang et al. (2025). Briefly, 20 mg of dried plant material was weighed and transferred into a 10-mL polypropylene centrifuge tube. Then, 5 mL of 1 mol·L^−1^ sodium hydroxide (NaOH) was added, and the mixture was shaken manually for 10 seconds before incubation in a preheated hot block at 120 ℃ for 1 h. After incubation, the tubes were cooled to room temperature and centrifuged at 4,000 rpm for 10 min. A 2-mL aliquot of the supernatant was transferred to a new 10-mL tube, followed by the addition of 3 mL of potassium acetate solution to neutralize the pH to between 5.0 and 5.5. Subsequently, 5 mL of total ionic strength adjustment buffer I was added to standardize the ionic strength. The F^−^ concentration in the solution was quantified based on the millivolt potential measured by the ion-selective electrode, which follows the Nernst equation: *E* = *S* log *C* + B. Where *C* is the F^−^ concentration in mg/L, *E* is the measured a millivolt potential, *S* is the slope, and *B* is the reference potential. Calibration curves were constructed using F^−^ standards (0.03, 0.10, 0.30, 1.00, and 3.00 mg L^−1^) prepared in 1 mol·L^−1^ NaOH to mimic the sample matrix. These standards were analyzed at regular intervals alongside plant samples to ensure measurement accuracy. Fluoride concentrations were then calculated by regression of the measured potential against the calibration curve.

### Elemental concentrations determined with monochromatic X-ray fluorescence spectroscopy

The concentrations of magnesium (Mg), Al, silicon (Si), phosphorus (P), sulfur (S), chlorine (Cl), potassium (K), calcium (Ca), Mn, iron (Fe), nickel (Ni), copper (Cu), and zinc (Zn) in plant tissues were analyzed using monochromatic X-ray fluorescence spectroscopy (MXRF E-lite and JP500, Z-Spec Inc., USA). Dried plant samples were first ground to a fine powder using a batch mill. For measurements, 100 mg of shoot tissue or 20 mg of root tissue was weighed and placed into an XRF sample holder, then covered by a 4.0-μm polypropylene thin film (Chemplex Industries, Inc., USA). The excitation energy was 4.51 keV for the E-lite instrument and 17.48 keV for the JP500 instrument. The optimal measurement procedures followed the method described in Kahlon et al. (2024) and Zhang et al. (2026). Quality controls included certified NIST standards (NIST 1568b, 1570a, and 1573a). The measurement mode was selected based on the sample type.

### Elemental mapping of plant organ sections using low-energy synchrotron X-ray fluorescence analysis

Given the limitations of F detection in hydrated samples, including full self-absorption of escaping F X-rays and intense scatter from H_2_O and the potential overlap of F emission line at 0.677 keV and O emission line at 0.525 keV, we used freeze-drying preparation. The methodology followed the procedures described in previous studies (Vogel-Mikuš et al. 2014). Briefly, fresh samples were rapidly frozen in liquid propane, cryo-sectioned to 30-µm-thick sections at −20 °C chamber temperature in a CM3050 Cryostat (Leica, Bensheim, Germany) and freeze-dried for 3 days at −94 °C and 0.001 mbar (Labogene, Denmark) to preserve structural integrity. Sections were photographed with an Axioskop 2 MOT microscope equipped with an Axiocam MRc color digital camera (Carl Zeiss AG, Göttingen, Germany) using UV excitation (365 nm). Low-energy X-ray fluorescence (LEXRF) measurements were conducted using the TwinMic X-ray fluorescence spectromicroscope at the Elettra Synchrotron Radiation Facility in Trieste (Gianoncelli et al. 2016). The LEXRF experimental setup was configured in accordance with prior studies to ensure consistency and accuracy in elemental mapping (Tolrà et al. 2011). The samples were sandwiched between 2 Pioloform foils for LEXRF analyses. TwinMic is a soft X-ray microscopy beamline running in the 400- to 2200-eV energy range (Kaulich et al. 2009; Gianoncelli et al. 2021). For the present experiment TwinMic was operated in scanning transmission mode (STXM), where the samples are raster scanned across a microprobe delivered by a multilayer Au zone plate diffractive optics (diameter: 600 μm; outermost zone width: 50 nm). Some sample areas were analyzed at 1.78 keV to obtain optimal excitation of Al, Mg and Na, with a beam size of 2.05 μm in diameter and scanned with a 2-μm step size; other selected regions were analyzed at 1 keV, to get optimal excitation of F, with a beam size of 580 nm in diameter and scanned with a 500 nm step size. Samples were raster-scanned perpendicularly to the incoming monochromatic beam, while a fast readout CCD camera collected the transmitted X-rays, and an 8-silicon drift detector-based XRF system acquired the emitted X-ray fluorescence photons (Gianoncelli et al. 2013). The obtained absorption and phase contrast images (Gianoncelli et al. 2006) outline the morphological sample features at sub-micrometer length scales, while the simultaneous detection of the low energy µ-XRF correlates the elemental distribution to the morphology. Elemental distribution was obtained with PyMCA software (Solé et al. 2007), by deconvolving and fitting the XRF spectra. XRF maps were plotted with XRFitVis visualization tool (Kourousias et al. 2026).

### Elemental mapping of whole leaves using micro-particle-induced gamma-ray emission (micro-PIGE) and micro-particle-induced X-ray emission (micro-PIXE)

Element allocation mapping at whole leaf level was conducted in-air at the external beamline of the Microanalytical Centre of the Jožef Stefan Institute, Ljubljana, Slovenia. Fluorine distribution was determined by micro-PIGE and, simultaneously, K, Ca, Mn, and Fe distributions were determined using micro-PIXE. Proton beam focusing, and beam dose normalization were performed as described previously (Isaković et al. 2022). During analyses, proton beam with 3 MeV nominal energy, 5 nA current, focused to ∼50 × 50 µm^2^ size exited the accelerator beamline trough a 200 nm thick silicon nitride window to excite gamma and X-rays from the leaf samples placed vertically on a motorized computer controlled XYZ stage, which moved the samples laterally across the beam with a µm precision. For each leaf sample, 3-s dwell time was selected with a step size of 50 or 100 µm. Two detectors were used simultaneously. The first was a high purity Ge (HPGe) detector (P-type coaxial; ORTEC, USA; 40% relative efficiency), which recorded 2 F peaks: a smaller at 110 keV and a larger 197 keV and 2 Al peaks: the first at 843 keV and the second at 1,014 keV (Fig. S5). The intensity of the 197 keV peak (for F) and of the 1,014 keV peak (for Al) was obtained by fitting a single Gaussian line to the region of interest in the spectrum after subtracting the continuous background following the algorithm used for X-rays in XANTHO program (Šmit 2023). The set-up was calibrated by yield-dose ratio determined using CaF_2_ standard. Fluorine mass concentrations were calculated on the surface approximation with proton stopping power estimated for cellulose as target matrix and on negligible (±8%) deviation of proton current. Aluminum distribution is qualitative. The second detector was a Si(Li) detector (Princeton Gamma-Tech Instruments, New Jersey, United States) with an active area 30 mm^2^, 7.5 μm thick Be window, 138 eV FWHM resolution at 5.9 keV which recorded emitted X-rays (*i.e.* K, Ca, Mn and Fe) from the sample. The detector was equipped with a pinhole filter of 3 mm diameter composed of 0.05 mm Aluminum foil with 1-mm opening; in addition, an absorber of 50 μm Kapton was used. Micro-PIXE spectra were processed in XANTHO (Šmit 2023) and distribution maps were generated in PyMCA software. The K-means clustering was performed in Quasar (https://quasar.codes/) based on z-normalized data (Toplak et al. 2021).

### Scanning electron microscopy (SEM) with energy dispersive X-ray spectroscopy (EDS)

The freeze-dried tea plant sections (leaves, petals, and roots) prepared as described earlier were mounted onto Aluminum SEM stubs using carbon stickers. Subsequently, the substrates were sputter coated with 12 nm of tungsten to assure good conductivity (SCD 500, Leica, Vienna, Austria). We performed field emission SEM (Magellan 400, Thermo-Fischer/FEI, Eindhoven, the Netherlands) with an energy-dispersive X-ray spectroscopy (EDX) detector to validate and measure the elements C, O, Mg, Al, Ca and K in the freeze-dried sections. Images were captured with the secondary electron detector set at 2 kV and 13 pA. Elemental analysis of C, O, K, Mg, Ca, and Al was performed using EDX spectrometer (Xmax 50, Oxford Instruments, Abingdon, UK). For EDX the samples were analyzed at 10 kV.

### Statistical analyses

All statistical analyses were performed using GraphPad Prism 8.0.1. One-way and two-way ANOVA were used to evaluate differences among treatment groups, with *P* < 0.05 considered statistically significant. Data are presented as mean ± standard deviation (SD) from at least 3 independent biological replicates. Hierarchical cluster analysis was performed using the Wu Kong online platform (https://www.omicsolution.com/wkomics/main/). The raw data were first subjected to log_10_ transformation followed by mean normalization. Euclidean distance was used to calculate pairwise dissimilarities, and clustering was conducted using the complete linkage method. Principal component analysis (PCA) was conducted using Simca-P software (Umetrics, Sweden) in PCA mode to assess the data structure and variance distribution.

## Results

### Growth responses of tea plants in Al and F^−^ treatments

**Figure 1:**
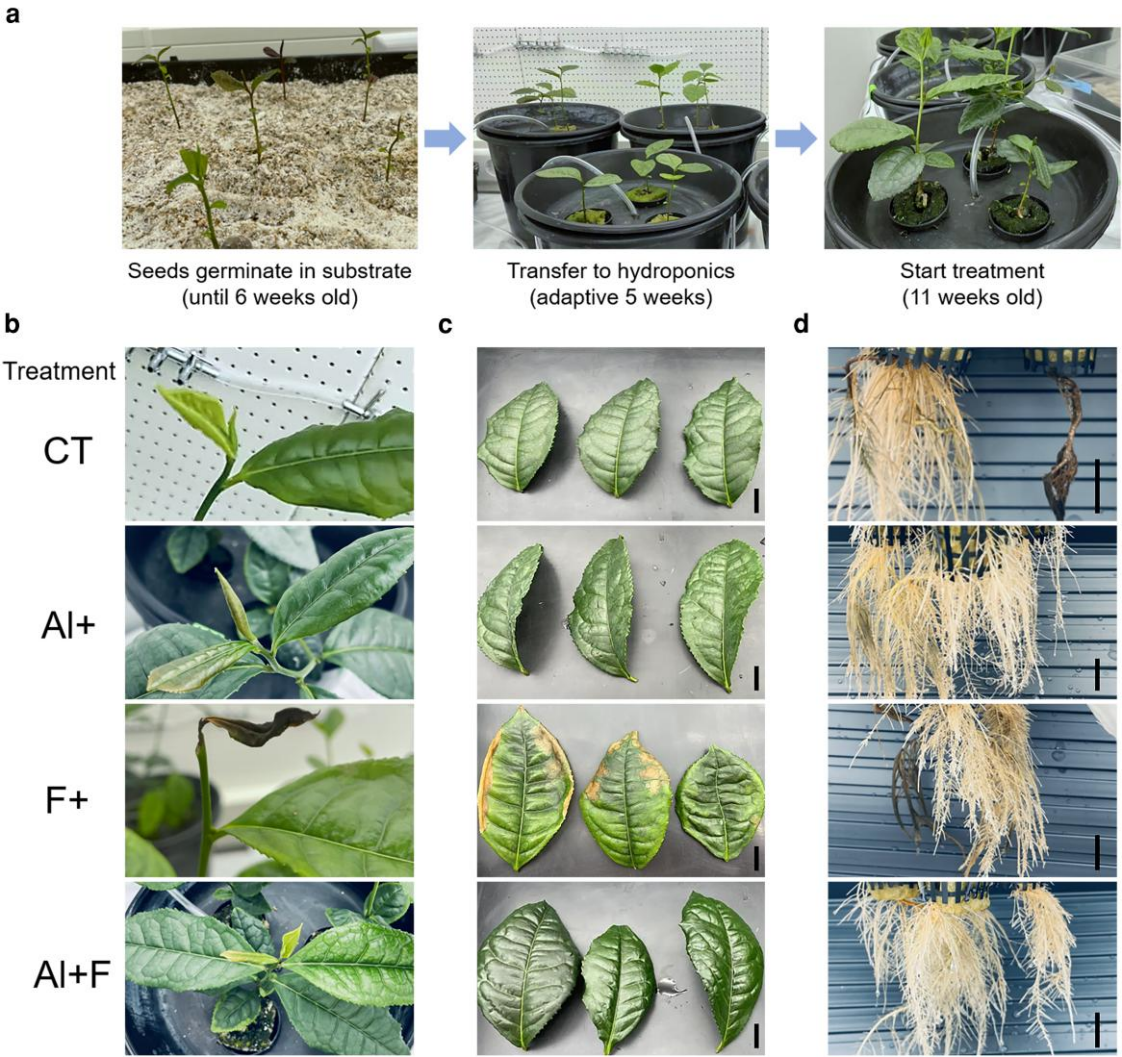
The effects of aluminum (Al) and fluoride (F^−^) on tea plants growth in the hydroponic conditions. **a)** Schematic diagram of growth of tea seedlings in the hydroponics system. **b–d)** Representative phenotypes of tea seedlings after 2 months of treatment: **(b)** tea buds, **(c)** mature leaves, **(d)** tea roots under different Al and F^−^ treatments. Abbreviations: Al+, AlCl_3_ treatment; Al + F, combined AlCl_3_ and NaF treatment; CT, control (pH 4.2); F+, NaF treatment. Each treatment included at least 9 seedlings (3 seedlings per pot). Scale bar = 1 cm.

Previous studies have shown that F^−^ (NaF ≥ 250 μM) significantly inhibits tea root growth—by more than 50%—in the absence of Al, whereas supplying these plants with Al (AlCl_3_ ≥ 250 μM) promotes root growth and alleviates F^−^ toxicity under hydroponic conditions after 40 days of treatment (Yang et al. 2016). In the present study, tea seedlings grown in a hydroponics system were treated with 250 μM NaF (F treatment) and 250 μM AlCl_3_ (Al treatment), or their combination (Al + F treatment), and phenotypic changes were recorded after 2 months of treatment and compared with control (CT) treatment (Fig. 1a). As shown in Fig. 1b, F treatment severely inhibited bud development, leading to bud death, while no significant differences were observed in the other treatment groups. In mature leaves, F treatment caused leaf crinkle, chlorosis, and margin necrosis, indicating that plants experienced F toxicity in this treatment. By contrast, the addition of Al in the Al + F treatment effectively alleviated these symptoms. Notably, the leaves of the Al + F group appeared even greener than those of the Al and CT treatments (Fig. 1c). In roots, both the CT and F treatments resulted in poor new root development and visible symptoms of phytotoxicity, whereas vigorous, healthy root growth was observed in the Al and Al + F treatments (Fig. 1d). Overall, our results were consistent with the previous studies, suggesting that Al plays an essential role in tea plant growth and that F^−^ tolerance in tea is largely dependent on the presence of Al.

**Figure 2:**
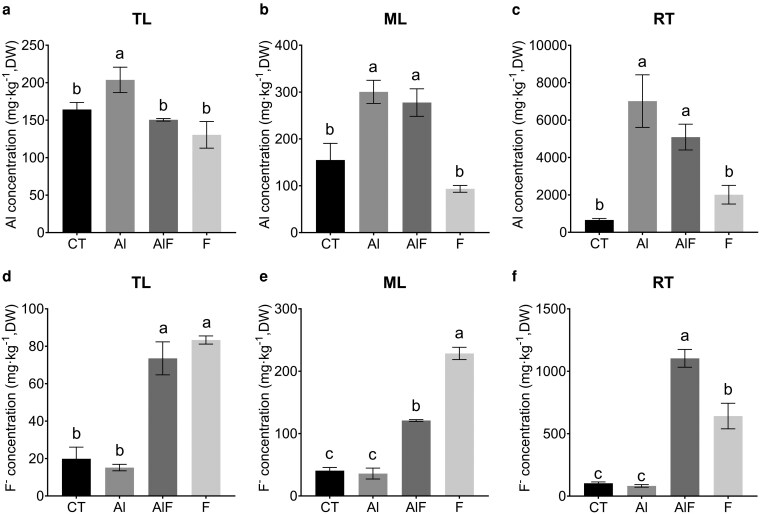
The concentration of aluminum (Al) and fluoride (F^−^) in tea plants grown under control (CT), Al and F^−^ treatments, and their combination (AlF) at pH 4.2. **a–c)** The concentration of Al in different tissues: **(a)** tender leaves (TL); **(b)** mature leaves (ML); and **(c)** roots (RT). **d–f)** The concentration of F^−^ in different tissues: **(d)** TL; **(e)** ML; and **(f)** RT. Abbreviations: Al, AlCl_3_ treatment; AlF, combined AlCl_3_ and NaF treatment; CT, control (pH 4.2); F, NaF treatment. Treatment concentrations are given on Fig. 1. Data are presented as mean ± standard deviation; Each treatment included at least 9 seedlings (three seedlings per pot). Different lowercase letters above bars indicate statistically significant differences among multiple groups (*P* < 0.05, One-way ANOVA followed by Tukey's multiple comparison test).

To investigate the underlying mechanism by which Al mitigates F^−^ toxicity, we quantified Al and F^−^ concentrations by using monochromatic X-ray fluorescence (MXRF) and F^−^ ion-selective electrode (ISE) in tender leaves (TL), mature leaves (ML), and roots (RT) for the CT plants and for the 3 different treatments. Aluminum concentrations ranged from 150 to 203 mg·kg^−1^ in TL, 155 to 300 mg·kg^−1^ in ML, and 660 to 7015 mg·kg^−1^ in RT (Fig. 2a–c). As expected, Al concentrations in the CT and F treatments were significantly lower than those in the Al treatment. Notably, the Al and F^−^ concentrations in plants before transfer to hydroponic culture were comparable to those observed in the CT treatment (Table S1). The addition of Al (Al + F treatment) significantly increased Al accumulation in TL, ML, and RT. Fluoride concentrations ranged from 15 to 73 mg·kg^−1^ in TL, 36 to 121 mg·kg^−1^ in ML, and 81 to 1104 mg·kg^−1^ in RT, respectively. In TL and ML, F^−^ concentration decreased following Al addition (Fig. 2d and e), while it significantly increased in RT in the Al + F treatment (Fig. 2f). These results suggest that Al enhances Al accumulation in both roots and leaves while reducing the translocation of F^−^ from roots to shoots.

### Tissue ionome dynamics in tea in response to Al and F^−^ treatments

**Figure 3:**
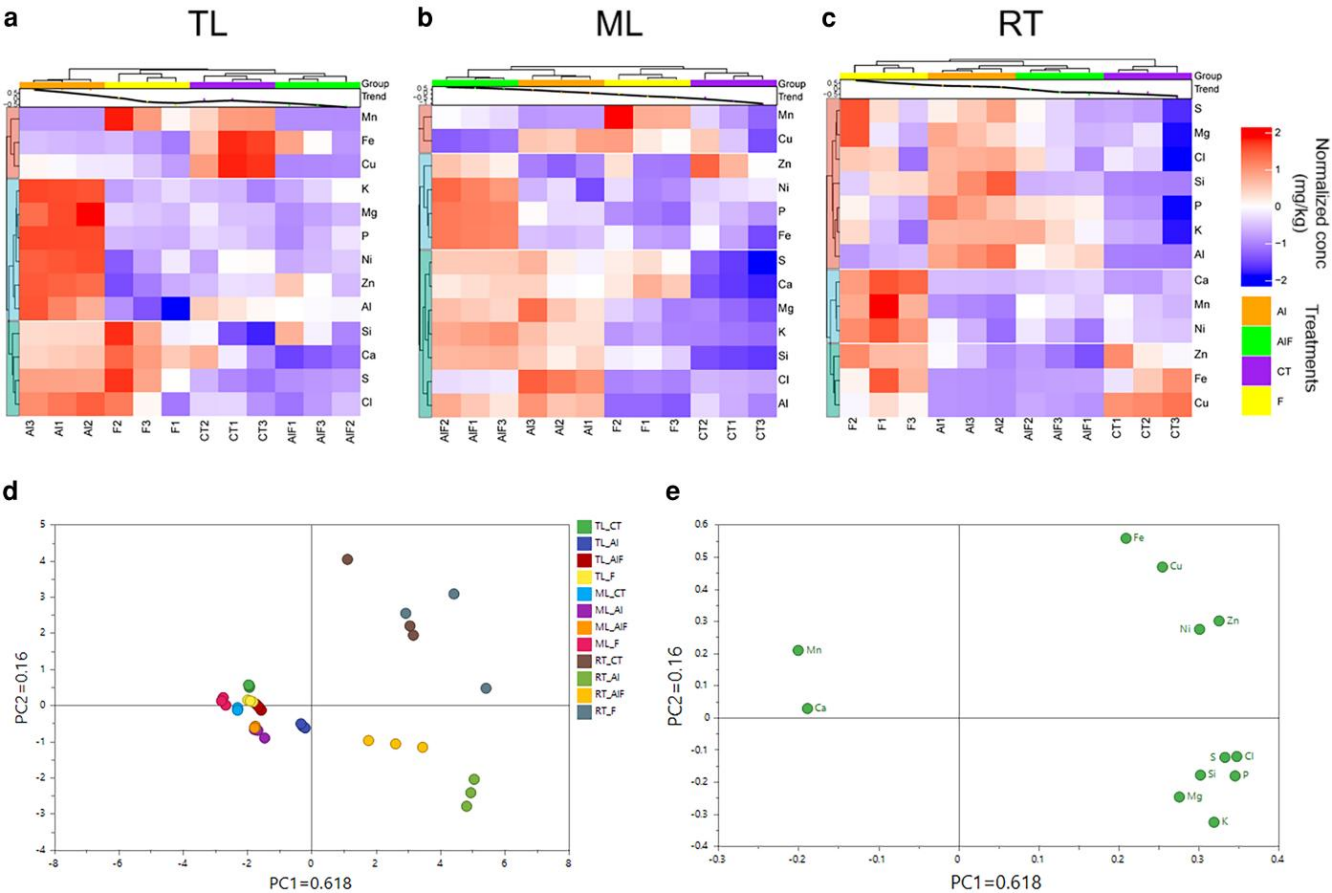
Effects of Al and F on the tea plant ionome under hydroponic conditions. **a-c)** Hierarchy cluster analysis of 12 elements in different tissues: **(a)** tender leaves (TL), **(b)** mature leaves (ML), and **(c)** roots (RT) under control (CT), Al and F^−^ treatments (F) and their combination (AlF), all at pH 4.2. Treatment concentrations are given in Fig. 1. Element concentrations were log_10_-transformed by row. Both rows and columns were clustered using Euclidean distance and complete linkage. **d, e)** Principal component analysis of the 12 elements in different tissues: **(d)** scored plot and **(e)** loading plot. Each treatment included at least 9 seedlings (3 seedlings per pot).

To further understand how Al alleviates F^−^ toxicity from an ionomic perspective, we quantified the concentrations of 12 elements in TL, ML, and RT, all of which are closely associated with plant development (Table S2). Hierarchical clustering analysis (HCA) revealed that in TL, the CT and Al + F treatment groups shared similar elemental composition, generally exhibiting lower concentrations than those under Al or F treatments (Fig. 3a). Compared with the CT treatment, we observed that Mg, P, K, Ni, and Zn concentrations increased in response to Al treatment, while Si, Cl, S, and Ca concentrations increased under both Al and F treatments. In both TL and ML, Mn concentration was increased by F treatment compared with Al + F treatment, suggesting Al may reduce F^−^-induced Mn toxicity. The Al + F treatment led to a general increase in elemental concentrations, often exceeding those seen under Al or F treatment alone (Fig. 3b). Clorine increased under Al treatment, and Cu increased under both Al and F treatments. Additionally, K and Mg specifically increased under both Al and Al + F treatments, suggesting Al supplementation may enhance K and Mg levels. In RT, F treatment resulted in a notable overall increase in elemental concentrations, while the Al and Al + F treatments clustered together, indicating a similarity in elemental compositions (Fig. 3c). Sulfur, Mg, Cl, P, K, and Si concentrations in RT increased under Al treatment, whereas Ca, Mn, and Ni increased in response to F treatment. Notably, Zn, Fe, and Cu levels decreased under both Al and Al + F treatments, suggesting that low pH and F^−^ exposure may induce Zn, Fe, and Cu toxicity in RT, while Al addition potentially mitigates these effects.

To assess the overall impact of Al and F^−^ on the ionomic profile at the whole-plant level, we performed principal component analysis (PCA). The first principal component (PC1) explained 61.8% of the total variance, primarily distinguishing between leaf and root samples, while the second principal component (PC2) accounted for 16% of the total variance, mainly separating treatment effects (Fig. 3d). Among tissues, the loading plot revealed that Mn and Ca mainly contributed to the leaf ionome; and other elements contributed to the roots, suggesting Al alleviates F^−^ toxicity primarily through root-mediated mechanisms. In roots, Fe, Cu, Mn, Ca, Ni, and Zn mainly contributed to CT and F^−^ treatments, whereas S, Cl, Si, P, Mg, and K were strongly associated with Al and Al + F treatments (Fig. 3e). Together with the HCA results, these findings suggest that Al alleviates F^−^-induced elemental disorders in leaves and roots.

### Elemental distribution in whole tea leaf

**Figure 4:**
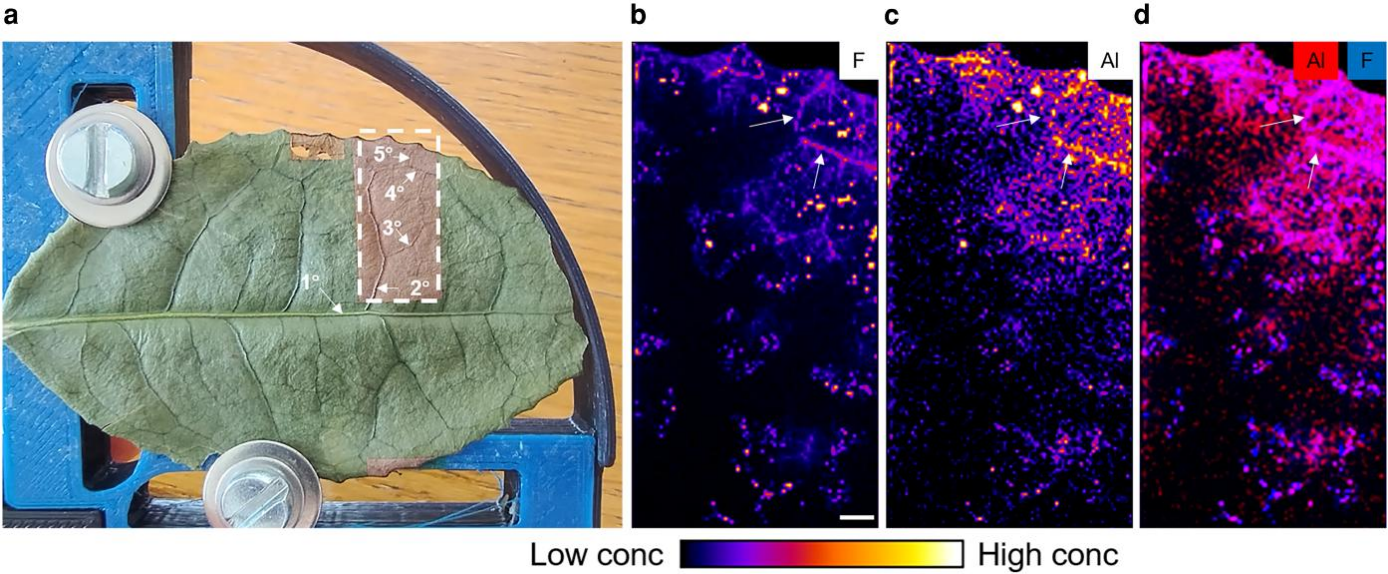
Distribution of aluminum (Al) and fluorine (F) in whole mature leaves as revealed by micro-PIGE mapping. **a)** Dehydrated tea leaf sample used for analysis. 1° to 5° indicates first to fifth order leaf veins. **b–c)** Elemental maps of the **(b)** F and **(c)** Al. **d)** Colocalization analysis of Al and F. Arrows indicate a leaf vein. Scale bar = 1 mm.

Previous studies using ^19^F NMR have shown that the addition of Al markedly diminished the peak of free F in cell saps, accompanied by the appearance of Al-F complexes in the leaves, thereby reducing F^−^ phytotoxicity in tea plants (Nagata et al. 1992, 1993; Yang et al. 2016). However, there has been no direct evidence confirming the *in situ* interaction between Al and F within plant tissues. In this study, we first analyzed their spatial distribution in whole tea leaves using micro-PIGE (Fig. 4a). This revealed that F is primarily localized in the interveinal areas near the leaf margin, with a distinct accumulation pattern along the fourth- and fifth-order veins (Fig. 4b). Similarly, Al predominantly accumulated in the leaf margin and the same lower-order veins (Fig. 4c). In contrast, K was mainly distributed in the second- and third-order veins and the leaf margin; Ca was concentrated in interveinal regions; and Mn and Fe appeared evenly distributed across the leaf surface (Fig. S1). To further investigate the spatial correlation of Al and F, we overlaid the elemental maps and observed strong colocalization of Al and F signals within the vein regions (Fig. 4d). Additionally, K-means clustering for all detected elements revealed that Al and F were grouped together, exhibiting highly similar distribution patterns (Fig. S2). Notably, all F hotspots co-occurred with Al, whereas not all Al-rich regions contained F, consistent with their differing concentration levels.

### Elemental distribution in tea plant leaf cross-sections

**Figure 5:**
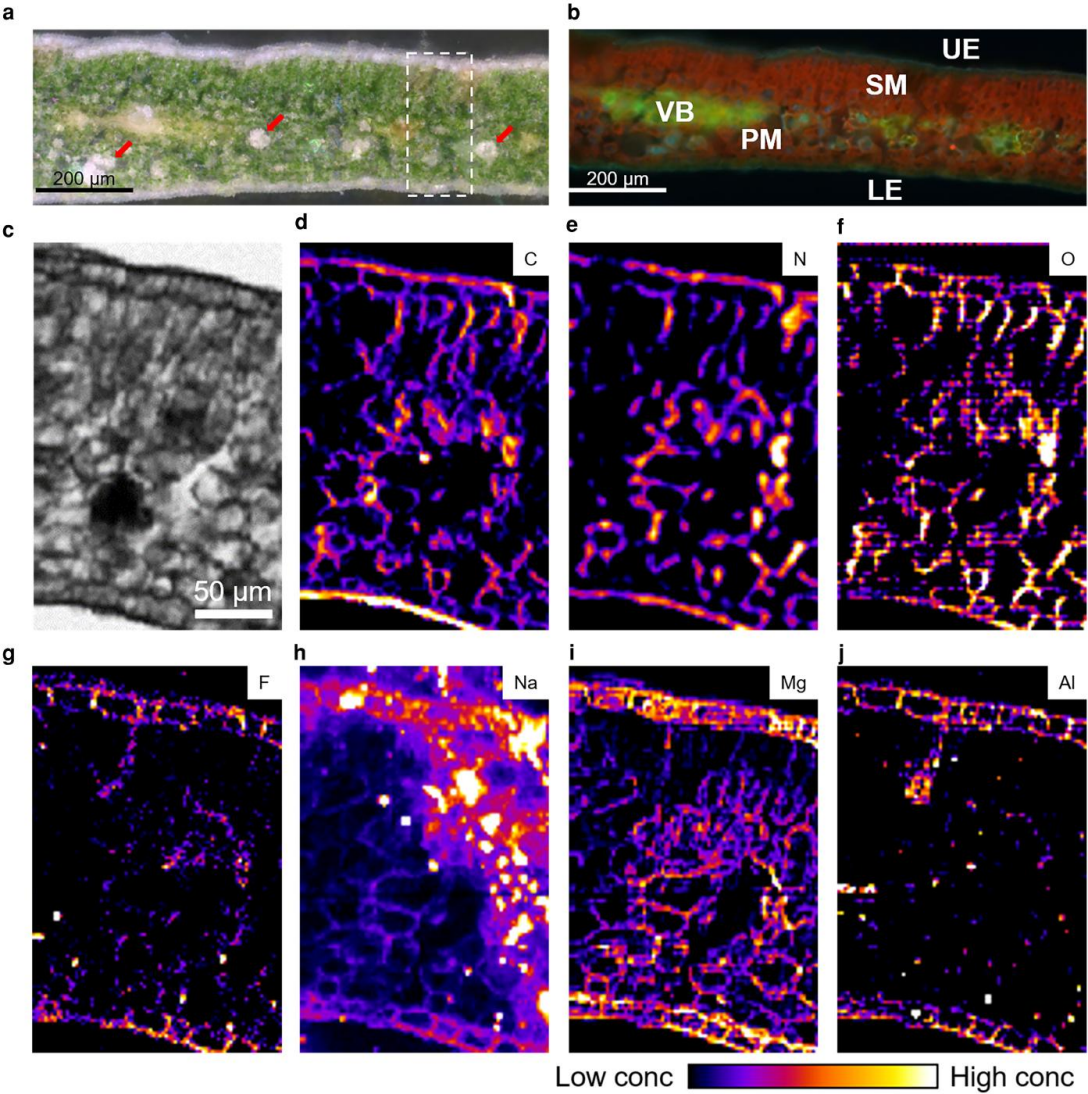
Elemental distribution in tea leaves. **a, b)** Cross-sectional images of leaf lamina: **(a)** bright-field image showing tissue structure and **(b)** fluorescence image highlighting auto-fluorescent features, including lower epidermis (LE), palisade mesophyll (PM), spongy mesophyll (SM), upper epidermis (UE), and vascular bundle (VB). **c–j)** Absorption and elemental maps of the cross-section: **(c)** absorption image; **(d)** carbon; **(e)** nitrogen; **(f)** oxygen; **(g)** fluorine; **(h)** sodium; **(i)** magnesium; **(j)** aluminum. Arrows indicate calcium-oxalate crystals.

In leaf cross-sections observed under bright-field microscopy, abundant chloroplasts were visible in both palisade mesophyll (PM) and spongy mesophyll (SM) cells. Additionally, Ca-oxalate crystals were distributed throughout the SM layer (Fig. 5a). Under fluorescence microscopy, vascular bundles (VB) were clearly observed within the SM (Fig. 5b). The scanning electron microscopy (SEM) analysis shows that the upper Ep, PM, and SM cells are well-preserved, the chloroplasts were abundant and distributed in both the PM and SM cells (Fig. S3). Using LEXRF, we found that C, N, and O were evenly distributed across the cross-section and mainly followed the cellular contours (Fig. 5c–f). Similarly, Na and Mg were evenly distributed in both the adaxial Ep cells and mesophyll cells, with Mg showing a relatively stronger signal in the Ep cells (Fig. 5h and i). The SEM-EDS analysis also confirmed that Mg is mainly distributed in the epidermal cells (Fig. S4). Both F and Al localized in the extracellular region of the Ep cells, and several co-localized hotspots were also observed within the mesophyll layer (Fig. 5g and j). K-means clustering analysis revealed that F and Al were grouped together in the extracellular regions of Ep cells (Fig. S5).

**Figure 6:**
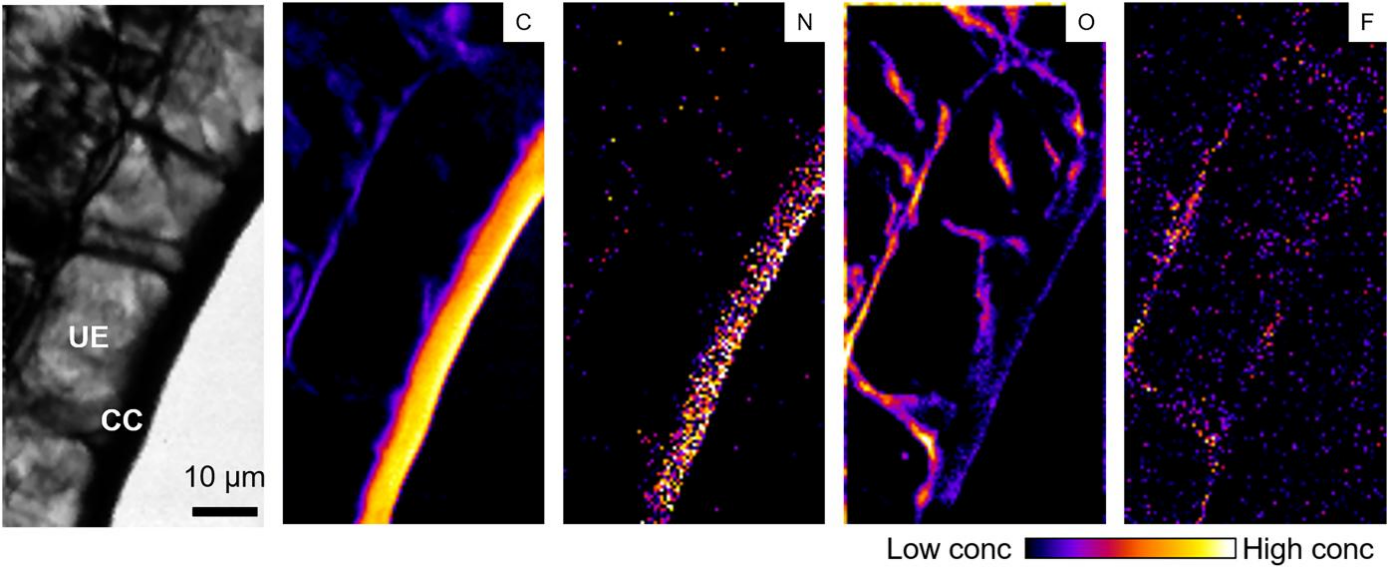
High-resolution elemental distribution in tea leaves. Absorption image of cross-sectional images of leaf lamina (CC, cuticle; UE, upper epidermis) together with the elemental distribution of carbon, nitrogen, oxygen, and fluorine.

To further investigate the localization of F in the epidermal cells, the excitation energy was reduced from 1.7 to 1 keV to better detect F. As shown in Fig. 6, C and N were predominantly accumulated in the cuticle of Ep cells, whereas O was distributed across both intra- and extracellular spaces. Compared with C, N, and O, the F signal was relatively weak; however, hotspots were observed along the cell contours of epidermal cells, suggesting that F is localized in the extracellular regions of these cells.

### Elemental distribution in tea plant petiole and midrib cross-sections

**Figure 7:**
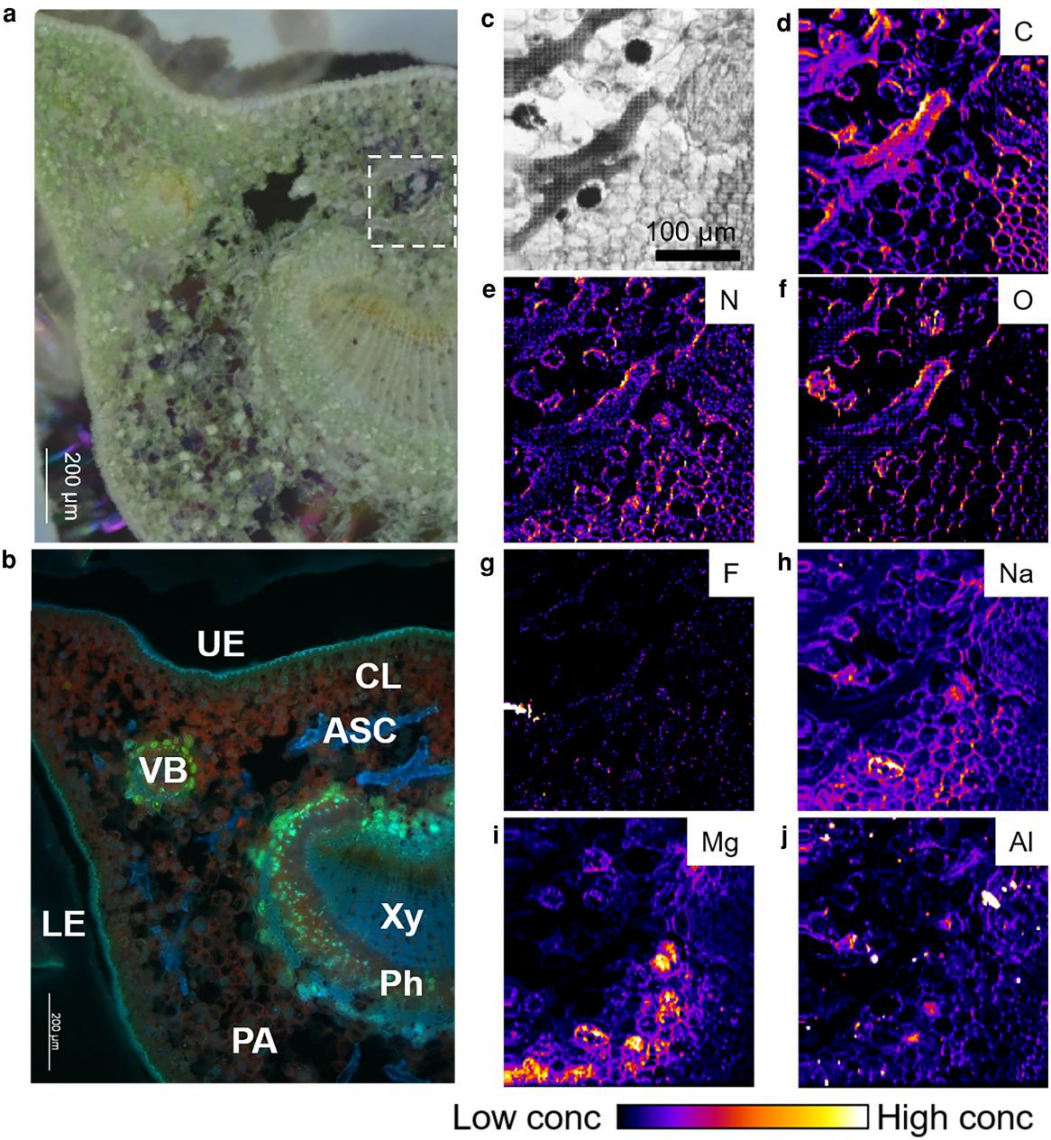
Elemental distribution in cross-section of tea plant petiole. **a, b)** Cross-sectional images of petiole: **(a)** bright-field image showing anatomical structure; **(b)** fluorescence image highlighting auto-fluorescent features, including astrosclereid (ASC); collenchyma (CL), lower epidermis (LE), parenchyma (PA), phloem (Ph), xylem (Xy), upper epidermis (UE), and vascular bundle (VB). c-j) Absorption and elemental mapping of the petiole cross-section: **(c)** absorption image; **(d)** carbon; **(e)** nitrogen; **(f)** oxygen; **(g)** fluorine; **(h)** sodium; **(i)** magnesium; **(j)** aluminum.

Previous studies have reported that Al exists in different chemical forms depending on tissue type: Al-catechin or Al-F complexes in leaves (Nagata et al. 1992), Al-citrate in xylem (Xy) sap (Morita et al. 2004), and Al-oxalate or Al-F complexes in roots (Morita et al. 2008). To investigate whether elemental speciation varies among different tissues, we analyzed cross-sections of the petiole and midrib. In the petiole, anatomical analysis revealed an oval-shaped cross-section with lateral projections toward the adaxial surface. The vascular system was of the collateral type, forming a continuous structure resembling that of the leaf midrib (Fig. 7a). Under autofluorescence microscopy, vascular bundles (VB) were clearly observed with the Xy and phloem (Ph) of the midvein (Fig. 7b). Notably, several astrosclereids (ASC) were embedded within the collenchyma (CL) layer. Elemental mapping showed that C, N, and O were highly accumulated in the ASC and in the apoplastic space of parenchyma (PA) cells (Fig. 7c–f). In contrast, F, Na, Mg, and Al were not allocated to the ASC (Fig. 7g–j). Specifically, the signals of F and Al did not exhibit colocalization pattern in petiole and midrib.

**Figure 8:**
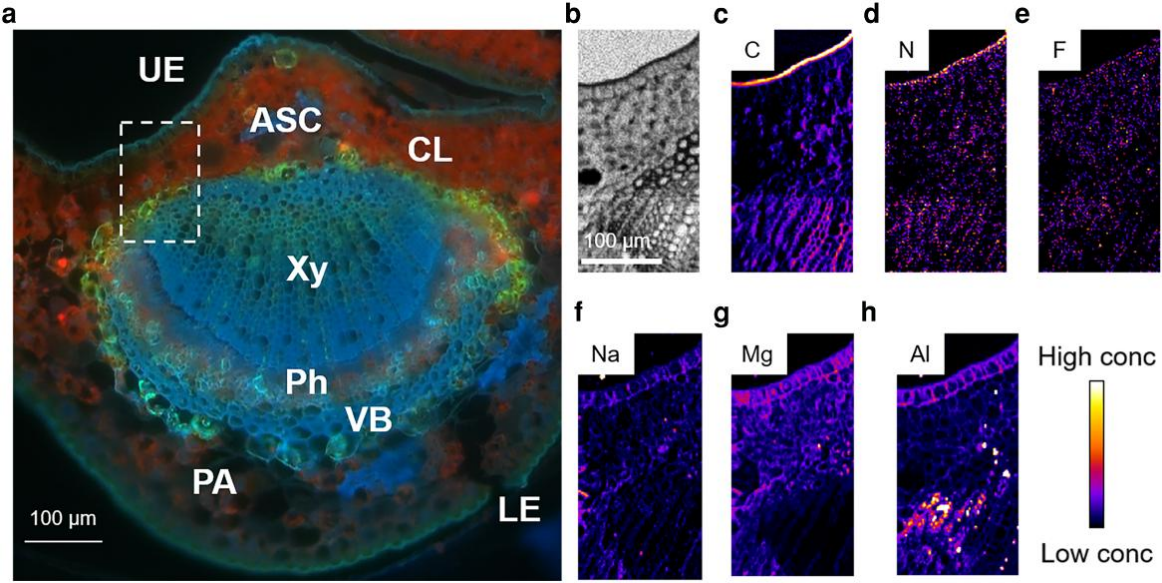
Elemental distribution in cross-section of tea plant midrib. **a)** fluorescence image highlighting autofluorescent features, including astrosclereid (ASC); collenchyma (CL), lower epidermis (LE), parenchyma (PA), phloem (Ph), xylem (Xy), upper epidermis (UE), and vascular bundle (VB). **b–h)** Absorption and elemental mapping of the midrib cross-section: **(b)** absorption image; **(c)** carbon; **(d)** nitrogen; **(e)** fluorine; **(f)** sodium; **(g)** magnesium; **(h)** aluminum.

To further validate the elemental distribution observed in the petiole, we analyzed the cross-section of the midrib. As shown in Fig. 8a, the vascular structure in the midrib was larger and more developed than that in the petiole. Consistent with the results from the leaf lamina, C and N were predominantly accumulated in the cuticle of the upper epidermis (Fig. 8b-d). F was distributed throughout the upper epidermis (UE), CL, and Xy (Fig. 8e). Sodium and Mg were highly concentrated in the UE and CL (Fig. 8f and g). Aluminum was localized in the extracellular space of the UE and, notably, was abundantly present in the Xy (Fig. 8h). However, no significant co-localization of Al and F was observed, confirming that Al-F complex is not the predominant chemical form responsible for the translocation of Al and F in tea plants.

### Elemental distribution in tea plant root cross-sections

**Figure 9:**
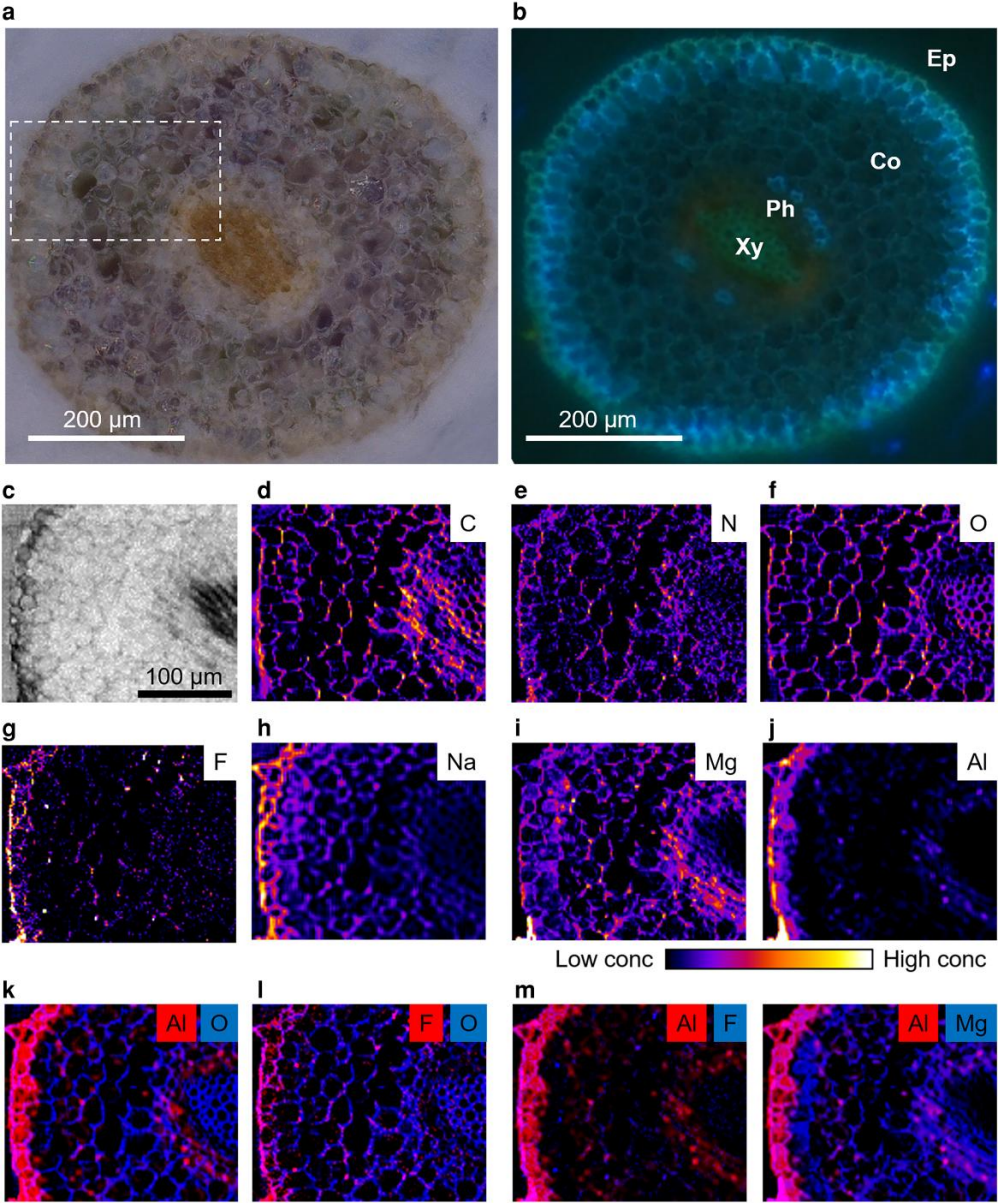
Elemental maps of Al, F, and other elements in cross-section of tea plants roots. a, **b)** Cross-sectional images of root: **(a)** bright-field image showing anatomical structure; **(b)** fluorescence image highlighting auto-fluorescent features, including cortical (Co), epidermis (Ep), phloem (Ph), and xylem (Xy). c–j) Absorption and elemental mapping of the root cross-section: **(c)** absorption image; **(d)** carbon; **(e)** nitrogen; **(f)** oxygen; **(g)** fluorine; **(h)** sodium; **(i)** magnesium; **(j)** aluminum. k–n) Colocalization analysis of O, F, Mg, and Al: **(k)** Al and O; **(l)** F and O; **(m)** Al and F; **(n)** Al and Mg.

In roots, the major cell types observed included Ep, cortical (Co), Ph, and Xy cells (Fig. 9a and b). Elemental mapping revealed that C, N, and O were broadly distributed throughout the apoplastic spaces of all cell types (Fig. 9c–f). Sodium was predominantly localized in the apoplastic regions of Co cells, whereas Mg was primarily concentrated in the Ph cells (Fig. 9h and i). Both F and Al were mainly distributed in the Ep (Fig. 9g and j), and K-means clustering analysis also showed that they were clustered together (Fig. S6). Aluminum was specifically enriched in the Ph, as confirmed by the colocalization of Al and O maps (Fig. 9k). In contrast, F was exclusively localized to the extracellular space of Co cells (Fig. 9l). Overlaying Al and F maps further revealed that these 2 elements were colocalized in the extracellular of Co cells (Fig. 9m). Notably, when Al and Mg maps were overlaid, we observed strong colocalization signal in the Co and Ph cells, particularly within the Co cells (Fig. 9n). Taken together, these results suggest that while Al and F may co-localize in the Ep, Al and Mg also show a colocalization trend in the Co and Ph tissues in roots. These findings imply distinct roles and transport pathways for Al and F in tea plants.

## Discussion

### Aluminum alleviates fluoride-induced Mn toxicity in tea plants

Fluoride is widespread in the environment but is toxic to most plants and animals. Plants are readily exposed to F^−^ through air, water, and soil. While many plant species exhibit toxicity symptoms at F^−^ concentrations below 20 μg·g^−1^ dry weight, tea plants (*Camellia sinensis*) can tolerate concentrations more than 1,000 μg·g^−1^ without apparent phytotoxic effects (van der Ent et al. 2021). A previous study suggested that F^−^ toxicity symptoms are absent in tea plants grown in acidic soils due to the co-accumulation of Al, which mitigates the inhibitory effects of F^−^ on root and shoot growth (Yang et al. 2016). Our findings confirm this observation and provide additional evidence in support of this hypothesis. Fluoride treatment did not significantly alter Al concentrations in either roots or leaves (*P* > 0.05). In contrast, Al supplementation increased F^−^ accumulation in roots while decreasing its accumulation in leaves. This pattern may be explained by the formation of Al-F complexes in roots, which reduce the mobility of F^−^, or by Al-induced root growth, which enhances overall uptake of F^−^. As Al concentrations in plant tissues greatly exceed those of F^−^, the reciprocal effect of F^−^ on Al uptake appears negligible.

The symptoms observed in tea leaves exposed to F^−^, particularly necrosis in the buds and at the margins of mature leaves, are very similar to “tip burn” in lettuce, a well-documented physiological disorder associated with localized Ca deficiency in leaf tissues (Macias-González et al. 2019). Previous studies have demonstrated that F^−^ can bind Ca and form CaF_2_ within plant tissues, which disrupts Ca gradient (Weinstein and Davison 2004). In this study, we found that F^−^ treatment did not markedly reduce Ca concentrations in tea leaves, which is possibly because tea stores excess Ca in leaves in oxalate crystals. Moreover, Ca levels were notably increased in the roots. This suggests that F^−^ may inhibit Ca translocation from roots to shoots, resulting in a Ca deficiency response. Moreover, F^−^ treatment significantly elevated Mn concentration in both leaves and roots, indicating a strong correlation between Mn accumulation and F^−^ exposure. A typical Mn toxicity symptom, leaf crinkle (El-Jaoual and Cox 1998; Li et al. 2019), was observed in mature leaves under F^−^ treatment. Therefore, a possible interaction between Ca deficiency and Mn toxicity would be expected. Although this hypothesis remains speculative, future studies could investigate whether Ca deficiency directly contributes to Mn accumulation and explore the dynamic ionomic responses associated with F^−^ exposure.

The mechanism by which tea roots tolerate and accumulate high levels of Al under low pH conditions remains unresolved. Previous research has shown that Al localizes in the nuclei of root meristematic cells and suggested it is an essential role in tea root development by maintaining DNA integrity (Sun et al. 2020). Marques et al. (2025) reported that Al is an essential element for the growth of *Borreria latifolia*, as Al supplementation promoted plant growth. However, it should be noted that Al stimulates growth only in certain plant species and under low pH conditions. In our pilot experiment, we observed that tea seedlings grow slowly, but normally, without Al supplementation at pH 5.5. Therefore, the term beneficial element is more appropriate for Al, as suggested by Ma et al. (2023). We hypothesize that Al confers a specific biological function in tea roots, contributing to their exceptional tolerance. However, the underlying molecular mechanisms warrant further investigation.

### Tissue elemental dynamics under Al and F^−^ treatment

Ionomics is a powerful tool for examining physiological responses to changes in nutrient status and environmental stress (Baxter et al. 2008). A previous study reported that Al-induced alterations in the ionome of tea plants were not pH-dependent and that Al specifically promoted Mn accumulation in both leaves and roots (Yamashita et al. 2020). However, our findings do not support this pattern. In our study, Mn concentrations in all 3 tissues were lower under Al treatment compared with the control. This result is consistent with earlier reports in tea, soybean, and barley, where Al was shown to reduce Mn toxicity and Mn accumulation through an antagonistic effect on Mn uptake (Wang et al. 1997; Yang et al. 2009; Muhammad et al. 2016). Despite similar phenotypic outcomes, this discrepancy may result from differences in plant materials, treatment durations, or measurement methodologies.

In this study, we observed that F^−^ treatment significantly increased Mn concentrations in all tissues. Leaf margin necrosis is a well-documented symptom of F^−^ toxicity in plants, though the underlying mechanisms remain unclear (Weinstein and Davison 2004). Symptoms of Mn toxicity include rapid chlorosis and necrosis in leaf margin and tip, as well as distortion of leaves (crinkle leaf) (González and Lynch 1999). These observations raise the possibility that F^−^ toxicity induces localized Mn accumulation in leaves. Upon Al supplementation, this F^−^-induced Mn accumulation was substantially reduced, further supporting the notion that Al alleviates F^−^-induced toxicity.

In addition, we found that F treatment increased Ca and nickel (Ni) concentrations in roots, while the Al + F treatment significantly reduced their levels. Previous research suggests that F^−^ can complex with Ca, disrupting cell wall structure by altering cross-linking, ultimately weakening cell walls and making them prone to collapse (Weinstein and Davison 2004). Thus, F^−^ may induce Ca deficiency by forming insoluble CaF_2_. Notably, previous study reported the simultaneous hyperaccumulation of Ni, Mn, and Ca in the leaf trichomes of some Ni-hyperaccumulating *Alyssum* (now *Odontarrhena*) species (Broadhurst et al. 2004), a pattern highly reminiscent of the elemental distribution observed in this study. These findings suggest a potential regulatory relationship among these 3 elements under F^−^ stress.

### Spatial localization of F and Al in tea plants

Understanding the spatial distribution and chemical speciation of elements at cellular or subcellular resolution is essential for elucidating their physiological roles and interactions in plant tissues (Kaulich et al. 2009). Tea is a well-known Al hyperaccumulator (Chenery 1955). Numerous studies have examined in situ Al localization in tea leaves using a variety of technologies, including electron microprobe X-ray analysis (Matsumoto et al. 1976), energy dispersive X-ray (Carr et al. 2003), low-energy X-ray fluorescence (Tolrà et al. 2011), X-ray fluorescence microscopy (van der Ent et al. 2020), and particle-induced X-ray emission (Uomori et al. 2013; Pongrac et al. 2020). A consistent finding across studies is that Al predominantly accumulates in the cell wall of the upper epidermis. In this study, we also observed a consistent localization pattern of Al in tea leaves. Moreover, in root cross-sections, Al distribution was mainly concentrated in the epidermis and endodermis, exhibiting a characteristic “double-ring” pattern. A similar distribution pattern was reported by Mesjasz-Przybyłowicz et al. (2007), who found that Ni enrichment in the hyperaccumulating genotype of *Senecio coronatus* occurred primarily in the epidermis and cortex, with concentrations several times higher than in the phloem. In contrast, in the non-hyperaccumulating genotype, Ni was more abundant in the phloem than in the epidermis or cortex. In rice, a typical silicon (Si) accumulator, Si enrichment, occurs in the cell walls of epidermal and endodermal cells (Moore et al. 2011). The *Lsi1* gene, localized to the plasma membrane on the distal side of both exodermal and endodermal cells, regulates Si uptake and accumulation in rice (Ma et al. 2006). Based on these findings, it can be speculated that element-specific transporters may also be expressed in both the epidermal and endodermal regions of tea roots, controlling Al uptake and distribution. In the future, it would be of great interest to screen low-Al-accumulating tea varieties and identify the genes and mechanisms responsible for Al uptake in tea plants.

The positive correlation between F and Al concentrations in tea plants (Ruan and Wong 2001; Ruan et al. 2003) has led to the hypothesis that their spatial distributions may overlap (Takamaz-Nisancioglu and Davison 1988). However, due to the high attenuation of F characteristic X-rays, its detection via conventional X-ray-fluorescence-based techniques has been challenging. To date, proton-induced gamma-ray emission (PIGE) has been the only technique demonstrated to map F in biological samples. Using micro-PIGE, Yoshida et al. (2013) localized F to the epidermis of tea leaves and proposed that it was cytoplasmic rather than cell wall associated. However, the relatively low spatial resolution of this method (400 μm × 400 μm) limited the precision of their conclusions. Several studies have applied low-energy X-ray fluorescence (LEXRF) to investigate the distribution of light elements such as C, N, and O in tea plants (Tolrà et al. 2011; Pongrac et al. 2020), suggesting that this technique can also be used to map F in biological samples. In the present study, we successfully mapped F and found that it is mainly distributed in the epidermal cells of tea leaves, consistent with the observations of Yoshida et al. (2013). Furthermore, we observed a co-localization of Al and F in both leaves and roots, confirming that F accumulation in tea plants is associated with Al-F complex formation.

Sample preparation, particularly dealing with water content, is the most critical step for achieving high-spatial-resolution elemental mapping at the tissue or subcellular level (Zhao et al. 2014), as well as for obtaining data that best reflect the living state (van der Ent et al. 2018). Both frozen-hydrated and freeze-dried samples can be used for elemental microanalysis; however, whenever technically feasible, mapping should ideally be performed on frozen-hydrated samples, as dehydration unavoidably causes artifacts at the subcellular level. Although the overall cell structure may appear intact, elements may no longer remain in their original locations, and some cells may appear empty in elemental maps. Some such alterations in elemental distribution between frozen-hydrated and freeze-dried leaves have been found in *Noccaea praecox* (Vavpetič et al. 2015). However, due to strong self-absorption of escaping F fluorescent X-rays and intense scattering from H_2_O, which becomes the matrix of the hydrated sample as leaf tissue can contain up to 90% water, it would be technically challenging, if not impossible, to map F in hydrated samples. Therefore, we adopted the freeze-drying method for F mapping. We found that O signals largely followed cellular contours, likely due to dehydration artifacts, as O should be uniformly distributed within the cell. In the LEXRF analysis (Figs 5, 8, and 9), Mg was mainly localized in the extracellular regions (cell walls). This contrast with previous studies on frozen-hydrated tissues (Carr et al. 2003) and freeze-dried tissues (Pongrac et al. 2020), where Mg was clearly intracellular. In SEM-EDS analysis (Fig. S4); however, both K and Mg appear more uniformly distributed within the cells. This discrepancy suggests variability in the success of sample preparation. Nevertheless, as validated by several studies (Castillo-Michel et al. 2017), the freeze-drying protocol preserves tissue integrity sufficiently to distinguish different cell types. In this study, we conclude that the observed co-localization trend of Al and F in the epidermis of tea leaves and roots is biologically meaningful. Recently, cryonanoscale secondary ion mass spectrometry (CryoNanoSIMS) has emerged as a promising technique for high-resolution elemental imaging of cryo-preserved samples (Testerink and van der Ent 2025), enabling the visualization of subcellular distributions of Na in the root meristem cells of *Arabidopsis* and rice (Ramakrishna et al. 2025). Therefore, CryoNanoSIMS may represent an alternative method for mapping F in frozen-hydrated plant tissues.

### Potential transport and tolerance mechanism in tea plants

Al in tea leaves is thought to be complexed mainly with catechins, with a smaller fraction bound to organic acids or F (Nagata et al. 1991, 1993). However, catechins are predominantly localized in vacuoles rather than cell walls (Suzuki et al. 2003; Xu et al. 2016), raising questions about the presence of Al–catechin complexes in vacuoles (Morita et al. 2008). A recent study, employing ^19^F NMR spectroscopy, identified AlF^2+^ complexes in the leaf cell sap of tea plants treated with both Al and F^−^ (Yang et al. 2016). These findings suggest that Al-F complexes represent a major chemical form of Al in leaves, whereas the existence of Al-catechin complexes warrants further investigation.

Previous studies have shown that Al is transported from roots to shoots as an Al-citrate complex, while F primarily exists in free form in the xylem sap (Morita et al. 2004). Consistent with this, we observed Al accumulation in the xylem of the midrib and petiole, whereas F was more evenly distributed in these organs. The distinct localization patterns of Al and F in vascular tissues support the idea that long-distance transport of Al does not occur as Al-F complexes.

In tea roots, Al tolerance is largely attributed to detoxification via the formation of soluble and insoluble complexes. Al-oxalate (1:2 and 1:3) complexes have been identified as the major Al species in tea roots (Morita et al. 2008), similar to those in buckwheat (Ma et al. 2001). However, the presence of Al-F complexes in tea roots remains controversial (Nagata et al. 1993; Yang et al. 2016). In this study, we observed high levels of Al and F in epidermal cells, though their colocalization was not evident in the phloem or xylem. Due to the substantial difference in Al and F concentrations in roots, our localization data alone may not conclusively demonstrate the presence of Al-F complexes. Nevertheless, considering that organic acids and cell walls possess stronger Al-binding affinities than F, while Al is the strongest known ligand for F in planta and is present in higher abundance, Al-F complexation remains a plausible and likely mechanism for F^−^ detoxification in tea plants.

Aluminum showed strong colocalization with Mg in both Co and Ph tissues. This may indicate potential competition between Al and Mg for membrane transporters or metal-binding sites on enzymes (Bose et al. 2011). Supporting this observation, our ionomic analysis revealed that Al treatment significantly increased Mg concentrations in roots. This aligns with previous findings in *A. thaliana*, where the Al-resistance mutant *alr104* maintained higher Mg^2+^ level than Al-sensitive mutants *als5* and *als3* under low-pH and Al stress conditions (Bose et al. 2013). It suggests that Mg plays a crucial role in maintaining Al tolerance in tea roots and points to the possible conservation of Al-Mg regulatory mechanisms across species.

## Conclusions

This study demonstrates that Al alleviates F-induced elemental imbalances in tea plants, notably by reducing excessive Mn accumulation. Aluminum and F were found to be co-localized in the fourth- and fifth-order veins at the leaf margins, specifically within the cell walls of epidermal cells in both leaves and roots, but not in the xylem of the petiole, midrib, or roots. In our study, we reveal the spatial distribution of Al and F across whole leaves and various tissues in tea plants, a species known for hyperaccumulating both elements. These findings provide strong evidence supporting the role of Al-F complex formation as accumulation and detoxification mechanism for both elements in tea plants. Future studies should explore the molecular mechanisms underlying the uptake, translocation, and distribution of Al-F complexes, as well as how Al mitigates F^−^-induced ionomic disruptions.

## Supplementary Material

kiag077_Supplementary_Data

## Data Availability

Data will be made available on request.
