# Supplementary material for: Fluoride detoxification in tea plants depends on aluminum and localization in the epidermis
Source: Plant Physiol. 2026 Feb 21;200(4):kiag077. doi: 10.1093/plphys/kiag077 (PMC13108599; doi:10.1093/plphys/kiag077)
Supplement: kiag077_Supplementary_Data [file kiag077_supplementary_data.pdf]

Supplementary Data

Fluoride Detoxification in Tea Plants Depends on Aluminium and Localization in the Epidermis

Chenyu Zhang[1, 2], Paula Pongrac[3,4], Katarina Vogel-Mikuš[3, 4], Alessandra Gianoncelli[5], Valentina Bonanni[5], Matjaž Kavčič[4,6], Žiga Šmit[4,6], Zdravko Rupnik[4], Primož Vavpetič[4], Mark G.M. Aarts[1], Antony van der Ent[1*]

[1]*Laboratory of Genetics, Wageningen University and Research, Droevendaalsesteeg 1, 6708 PB Wageningen, The Netherlands.*

[2]*Key Laboratory of Biology, Genetics and Breeding of Special Economic Animals and Plants, Ministry of Agriculture and Rural Affairs, Tea Research Institute of the Chinese Academy of Agricultural Sciences, Hangzhou 310008, China.*

[3]*University of Ljubljana, Biotechnical Faculty, Jamnikarjeva 101, Ljubljana, Slovenia.*

[4]*Jožef Stefan Institute, Jamova 39, Ljubljana, Slovenia.*

[5]*Elettra–Sincrotrone Trieste, S.S. 14, km163.5 in Area Science Park, 34149, Trieste-Basovizza, Italy*

[6]*Faculty of Mathematics and Physics, University of Ljubljana, Jadranska 19, 1000 Ljubljana, Slovenia*

Corresponding author: Antony van der Ent (antony.vanderent@wur.nl)

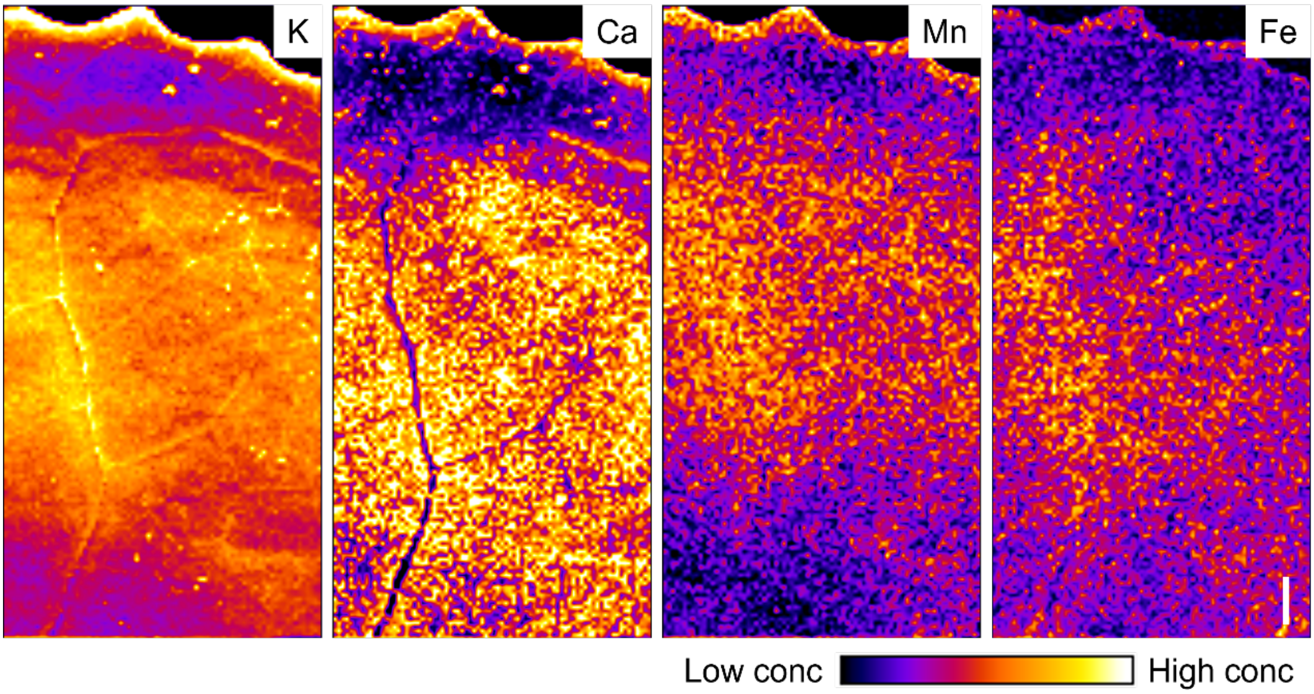

Supplementary Figure S1. Elemental maps of potassium (K), calcium (Ca), manganese (Mn), and iron (Fe). The leaf area was identical as in Fig. 4 of the main text, determined by micro-particle induced X-ray emission. Scale bar = 1 mm.

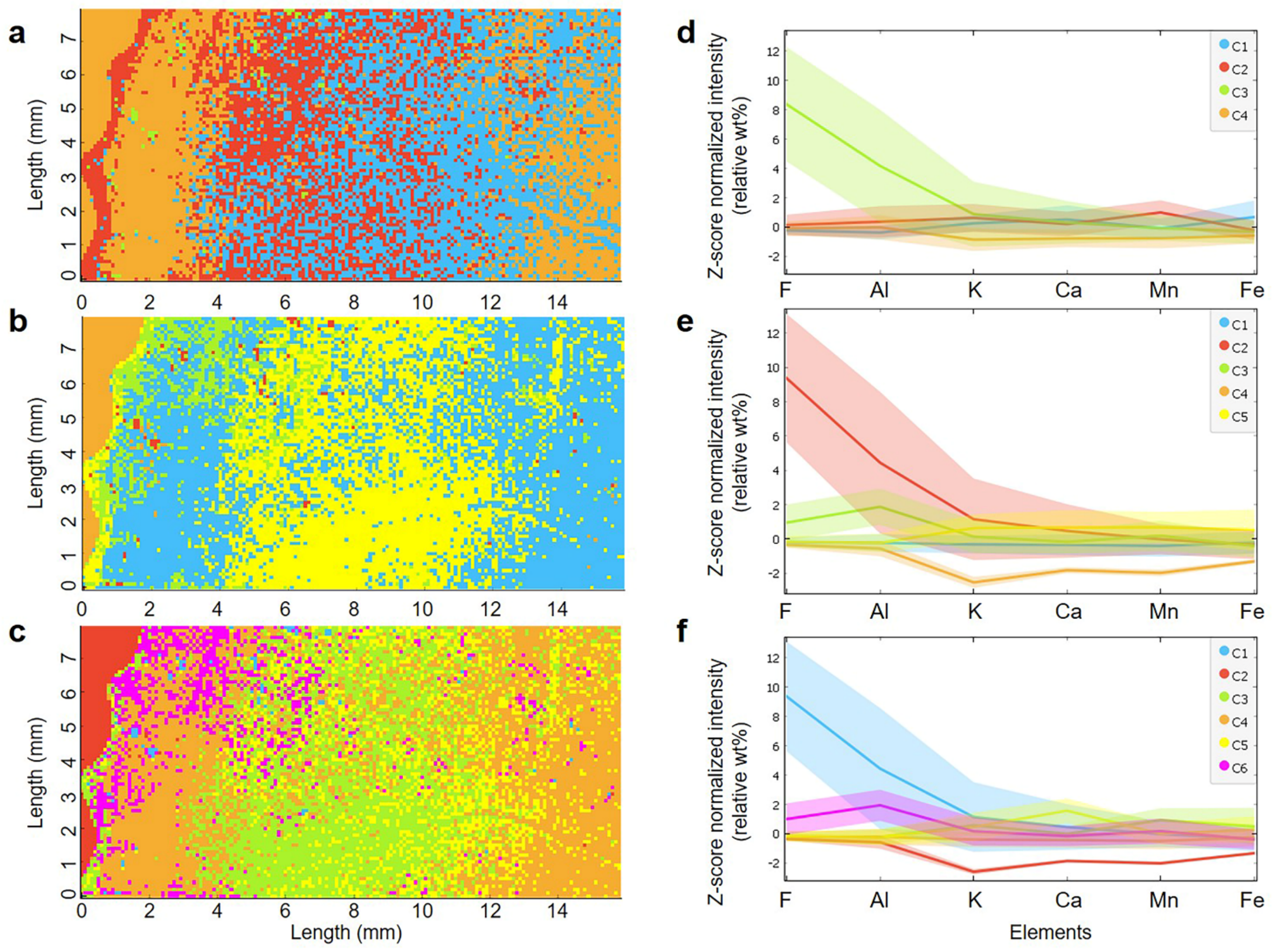

Supplementary Figure S2. K-means clustering of detected elements in whole leaf.

The distribution pattern of fluorine (F), aluminium (Al), potassium (K), calcium (Ca), manganese (Mn), and iron (Fe) were analyzed. (a–c) Elemental mapping under four (a), five (b), and six (c) clusters. (d–f) Corresponding clustering results for four (d), five (e), and six (f) clusters. Shaded areas indicate the 95% confidence interval for each element's concentration. Similar spatial distribution patterns among different elements are considered indicative of potential co-localization. Elements classified within the same cluster displayed similar spatial distribution trends, showing clearly co-occurrence of high or low signal intensities. Fluorine and Al showed strong co-occurrence of high signal intensities in Cluster 3 (in the four-cluster analysis), Cluster 2 (in the six-cluster analysis), and Cluster 1 (in the six-cluster analysis).

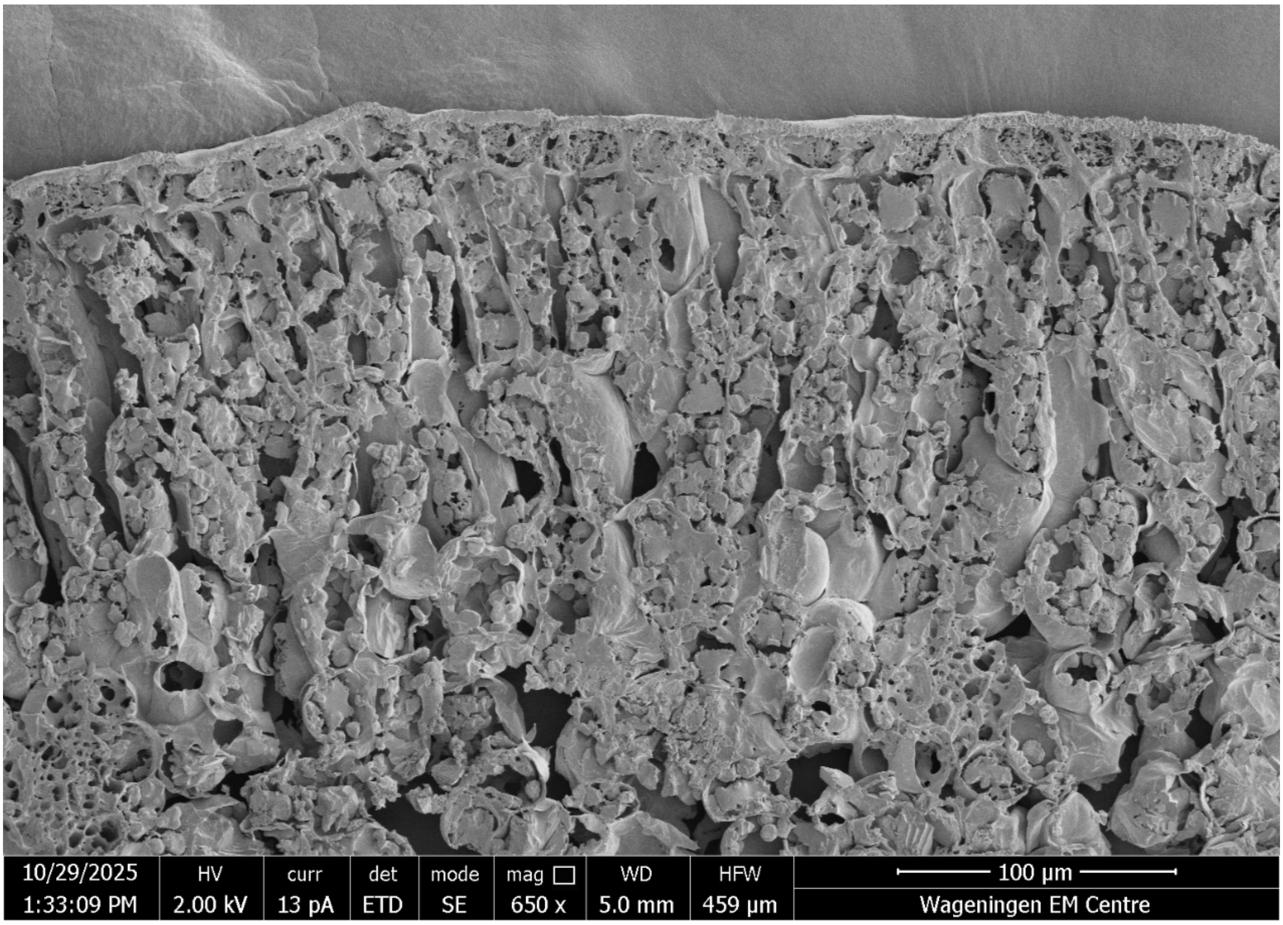

Supplementary Figure S3. Scanning electron microscopy analysis of a freeze-dried tea plant leaf cross-section.

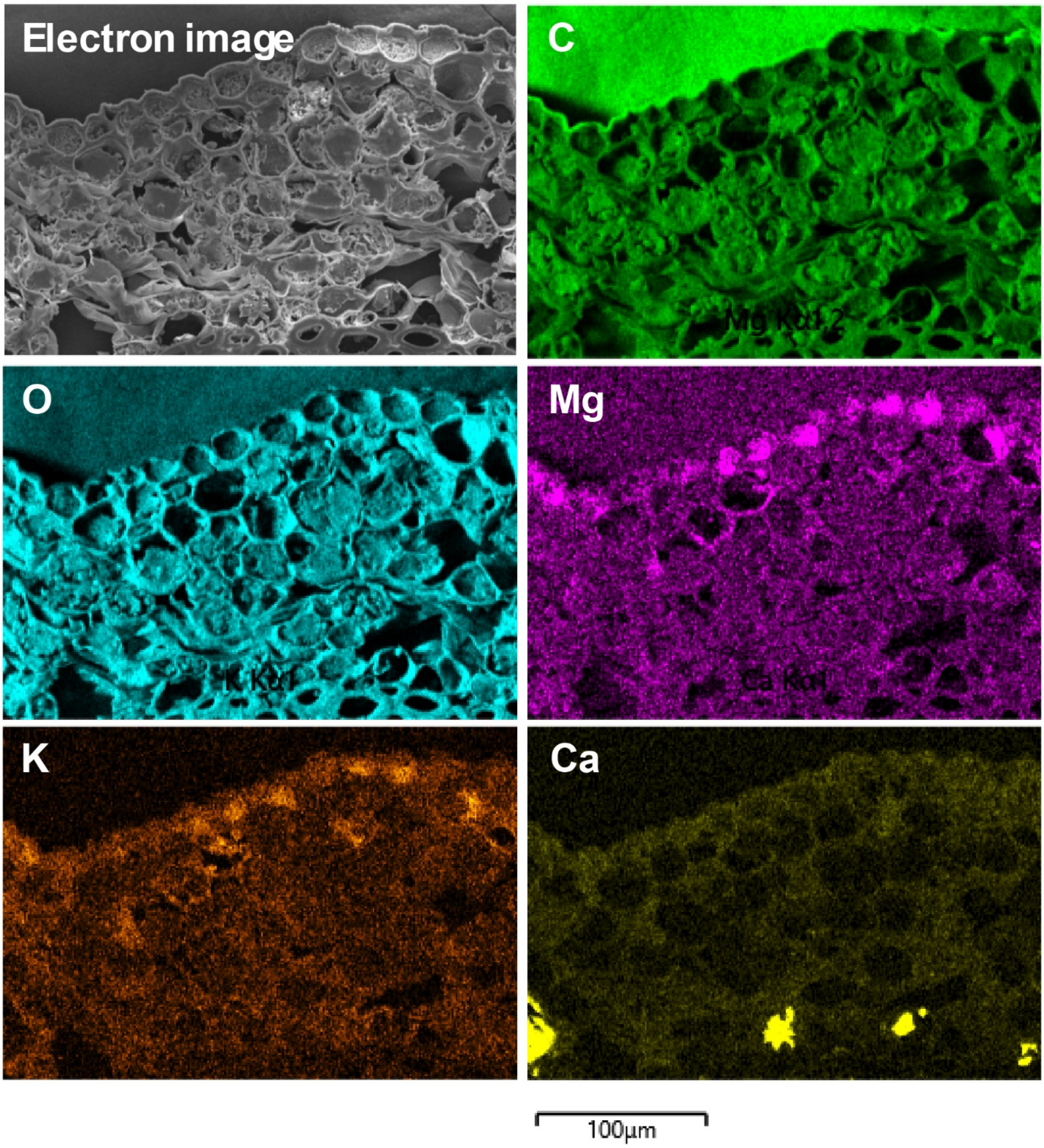

Supplementary Figure S4. Scanning electron microscopy-energy dispersive X-ray spectroscopy (SEM-EDS) analysis of a freeze-dried tea plant leaf cross-section. The distribution pattern of carbon, oxygen, magnesium, potassium, and calcium were analyzed.

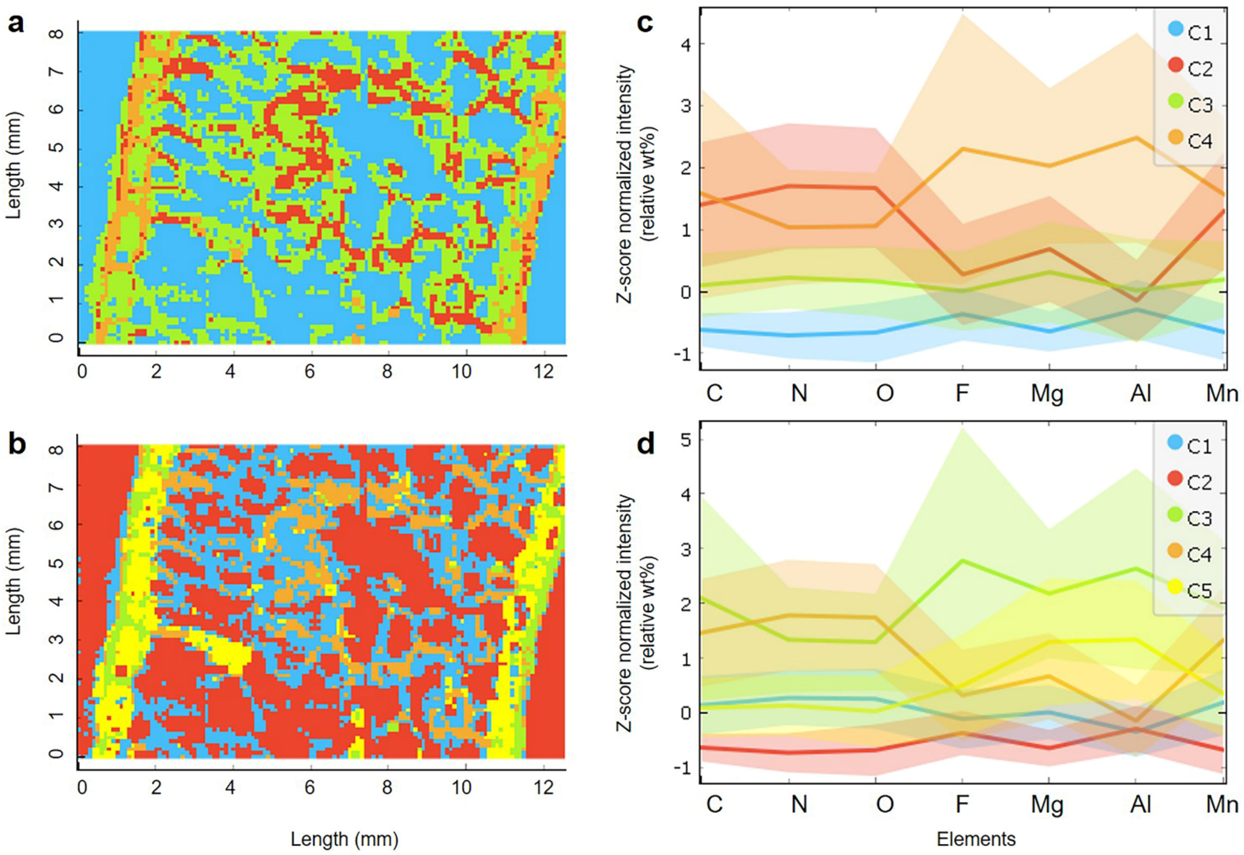

Supplementary Figure S5. K-means clustering of detected elements in leaf cross-section.

The distribution pattern of carbon (C), nitrogen (N), oxygen (O), fluorine (F), magnesium (Mg), aluminium (Al), and manganese (Mn) were analyzed. (a, b) Elemental mapping under four (a) and five (b) clusters. (c–d) Corresponding clustering results for four (c) and five (d) clusters. Shaded areas indicate the 95% confidence interval for each element's concentration. Similar spatial distribution patterns among different elements are considered indicative of potential co-localization. Elements classified within the same cluster displayed similar spatial distribution trends, showing clearly co-occurrence of high or low signal intensities. Fluorine, Mg, and Al showed strong co-occurrence of high signal intensities in Cluster 4 (in the four-cluster analysis) and Cluster 3 (in the five-cluster analysis).

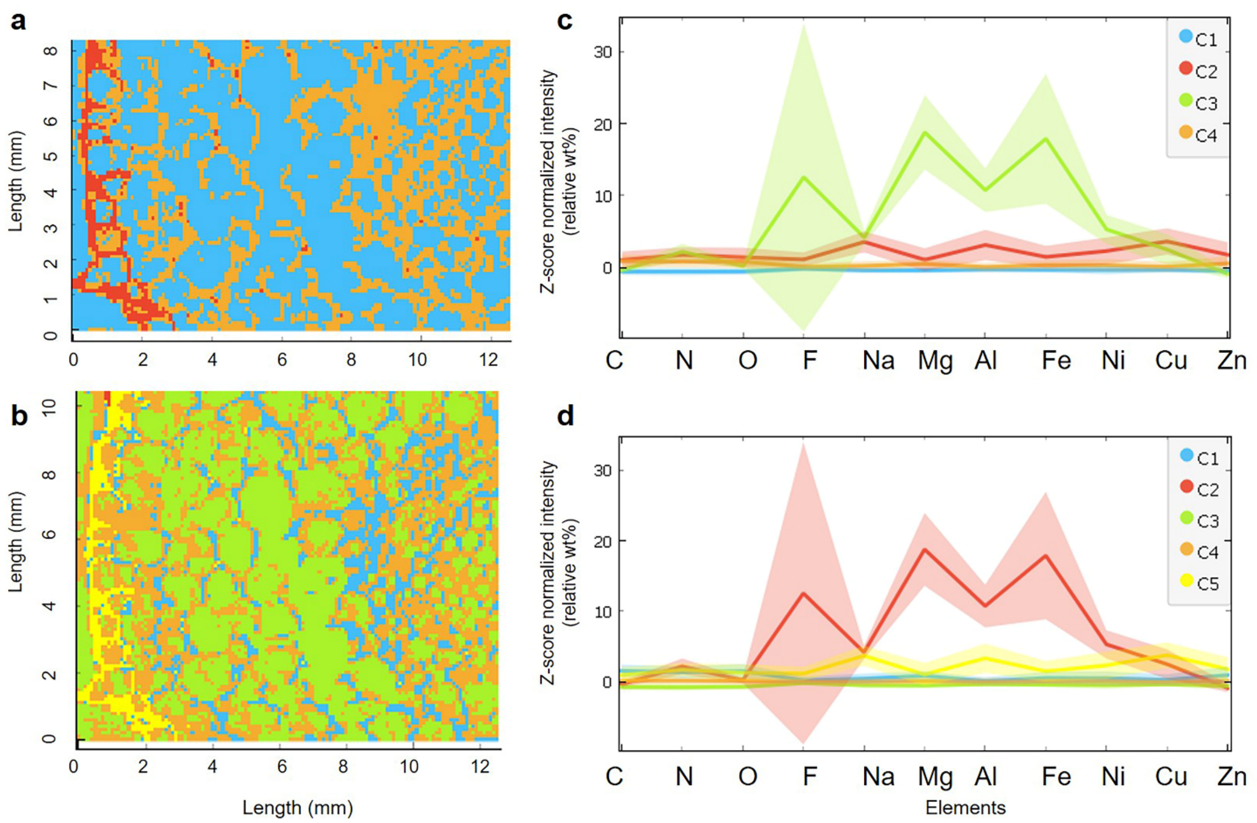

Supplementary Figure S6. K-means clustering of detected elements in root cross-section.

The distribution pattern of carbon (C), nitrogen (N), oxygen (O), fluorine (F), sodium (Na), magnesium (Mg), aluminium (Al), iron (Fe), nickel (Ni), copper (Cu), and zinc (Zn) were analyzed. (a-b) Elemental mapping under four (a) and five (b) clusters. (c–d) Corresponding clustering results for four (c) and five (d) clusters. Shaded areas indicate the 95% confidence interval for each element's concentration. Similar spatial distribution patterns among different elements are considered indicative of potential co-localization. Elements classified within the same cluster displayed similar spatial distribution trends, showing clearly co-occurrence of high or low signal intensities. Fluorine, Mg, Al, and Fe exhibited strong co-occurrence of high signal intensities in Cluster 3 (in the four-cluster analysis) and Cluster 2 (in the five-cluster analysis).

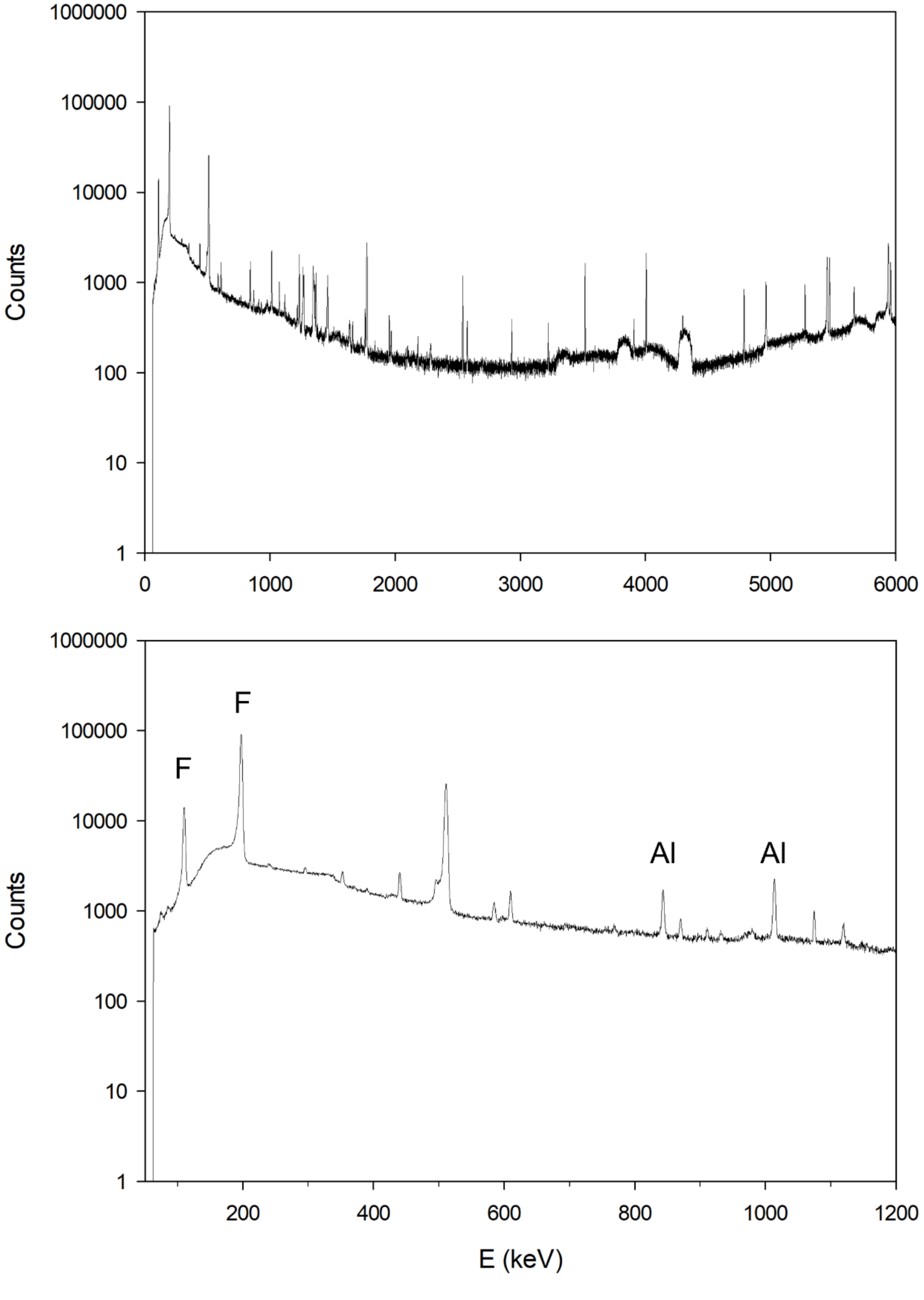

Supplementary Figure S7. Particle-induced gamma-ray emission spectrum.

The top panel shows the cumulative spectrum, and the bottom panel shows the fluorine (F)- and aluminium (Al)-relevant regions.

Supplementary Table S1. Aluminium and fluoride concentrations in leaves and roots of tea plants prior to transfer.

Samples	Elements (mg/kg, DW)	
	F	Al
Leaves-1	31.35	355.55
Leaves-2	25.65	456.82
Leaves-3	30.65	306.19
Roots-1	100.64	693.5
Roots-2	99.57	606.04
Roots-3	85.45	649.77

Note: Plants were grown for 6 weeks in a vermiculite and perlite mixture prior to hydroponic transfer.

Supplementary Table S2. Elemental concentrations in tender leaves (TL), mature leaves (ML) and roots (RT) of tea plants.

Samples	Elements (mg/kg, dry weight)											
	**Mg**	**Si**	**P**	**S**	**Cl**	**K**	**Ca**	**Mn**	**Fe**	**Ni**	**Cu**	**Zn**
TL-CT-1	700.59	53.44	2200	1650	186.04	12020	4420	236.34	106.72	1.14	5.56	22.60
TL-CT-2	696.60	50.76	2115	1585	168.17	11250	3940	236.97	105.95	1.15	5.52	22.42
TL-CT-3	692.60	48.08	2030	1520	150.30	10480	3460	237.60	105.18	1.15	5.48	22.23
TL-Al-1	1625	82.55	4240	2885	292.41	20325	5245	132.35	74.67	1.74	3.51	39.32
TL-Al-2	1740	82.95	4230	2870	304.77	20170	5340	132.12	73.89	1.76	3.36	38.55
TL-Al-3	1510	82.14	4250	2900	280.05	20480	5150	132.57	75.45	1.71	3.65	40.08
TL-AlF-1	515.71	88.54	1650	1680	133.67	11610	2730	125.83	94.48	0.87	2.49	30.38
TL-AlF-2	666.62	60.40	2380	1780	174.34	14230	3170	125.62	71.14	0.97	2.53	19.63
TL-AlF-3	591.17	74.47	2015	1730	154.01	12920	2950	125.73	82.81	0.92	2.51	25.01
TL-F-1	676.57	74.25	1950	2290	136.81	13110	5270	184.88	68.05	1.07	2.69	18.38
TL-F-2	761.99	104.95	2040	3450	290.17	11380	6380	287.16	74.84	0.62	3.35	10.83
TL-F-3	719.28	89.60	1995	2870	213.49	12245	5825	236.02	71.45	0.85	3.02	14.61
ML-CT-1	426.12	36.77	1465	1205	244.61	7505	3205	106.30	47.47	0.24	1.74	9.28
ML-CT-2	494.24	38.27	1660	1350	254.97	7920	3530	134.55	57.01	0.23	1.99	10.31
ML-CT-3	358	35.27	1270	1060	234.24	7090	2880	78.04	37.92	0.25	1.48	8.24
ML-Al-1	870.64	74.02	1660	1900	545.51	14410	6310	106.41	50.90	0.13	2.09	6.84
ML-Al-2	950.82	68.64	1650	2030	582.63	14410	6220	106.26	49.75	0.24	1.98	6.37
ML-Al-3	1190	75.20	1900	2300	624.46	15940	7200	103.23	50.61	0.21	2.0	6.73
ML-AlF-1	940.53	79.73	2755	2205	320.15	17475	6950	145.15	73.44	0.38	1.54	8.77
ML-AlF-2	975.01	79.17	2790	2200	311.10	16950	6720	154.06	74.14	0.40	1.51	9.09
ML-AlF-3	906.05	80.28	2720	2210	329.19	18000	7180	136.24	72.73	0.36	1.57	8.45
ML-F-1	610.05	64.29	1110	2110	195.35	9410	7810	205.38	47.25	0.17	1.91	6.68

ML-F-2	676.4	63.07	1220	2040	184	8760	6750	273.0	56.31	0.24	2.10	8.66
ML-F-3	475.9	58.25	1020	1970	178	8030	7320	203.8	46.24	0.18	1.86	6.64
RT-CT-1	1080	130.94	11340	3570	1330	24080	1170	89.51	3230	2.18	30.62	232.99
RT-CT-2	1260	117.59	10940	3390	1200	23010	1100	62.62	6230	1.90	31.51	172
RT-CT-3	353.5	109.78	4260	1870	392.5	6850	1740	52.14	8450	1.86	33.55	155.79
RT-Al-1	1865	564.83	24980	5015	1815	44165	1760	33.79	633.75	2.21	9.75	157.14
RT-Al-2	1600	766.53	21040	6280	1890	44910	1670	21.57	451.97	1.20	9.28	94.81
RT-Al-3	1732.5	665.68	23010	5648	1853	44538	1715	27.68	542.86	1.71	9.51	125.97
RT-AlF-1	1050	238.78	17830	3470	908	32330	2110	63.16	1090	1.28	9.69	66.00
RT-AlF-2	1340	216.97	18940	4830	1620	45250	1820	92.24	994.83	2.10	11.62	106.08
RT-AlF-3	1195	227.88	18385	4150	1264	38790	1965	77.70	1042.42	1.69	10.66	86.04
RT-F-1	1280	460.32	14020	5320	1670	25820	6390	218.20	10240	3.70	22.96	204.58
RT-F-2	2260	278.26	17720	7410	1720	38840	5080	148.61	4580	3.29	18.44	234.27
RT-F-3	1020	476.03	12220	4010	799.76	16760	6090	142.61	7070	3.20	20.04	203.70

Note: Plants grow hydroponically without treatments (control, CT) or treated with aluminium (Al), and fluorine (F) or both Al+F.

Supplementary Table S3. Composition of Hoagland solution.

Nutrients	Stock (g L^{-1})	Stock (M)	Element	0.5 × Hoagland (mL for 1 L solution)
KNO_3	101.1	1	N/K	3
$Ca(NO_3)_2 \cdot 4\ H_2O$	236.15	1	Ca/N	2
$NH_4H_2PO_4$	115.03	1	N/P	1
$MgSO_4 \cdot 7\ H_2O$	246.47	1	Mg/S	0.5
KCl	0.075	1	Cl	
H_3BO_3	1.546	25	B	
$MnSO_4 \cdot 4\ H_2O$	0.446	2	Mn	
$ZnSO_4 \cdot 7\ H_2O$	0.575	2	Zn	1
$CuSO_4 \cdot 5\ H_2O$	0.025	0.1	Cu	
$(NH_4)_6Mo_7O_{24} \cdot 4\ H_2O$	0.124	0.1	Mo	
Fe(Na)EDDHA	8.704	20	Fe/Na	1
